# Polyester urethane urea (PEUU) functionalization for enhanced anti-thrombotic performance: advancing regenerative cardiovascular devices through innovative surface modifications

**DOI:** 10.3389/fbioe.2023.1257778

**Published:** 2023-09-20

**Authors:** María A. Rodríguez-Soto, Natalia Suárez Vargas, María Ayala-Velásquez, Andrés M. Aragón-Rivera, Carlos Ostos, Juan C. Cruz, Carolina Muñoz Camargo, Seungil Kim, Antonio D’Amore, William R. Wagner, Juan C. Briceño

**Affiliations:** ^1^ Department of Biomedical Engineering, Universidad de los Andes, Bogotá, Colombia; ^2^ Group CATALAD, Instituto de Química, Universidad de Antioquia, Medellín, Colombia; ^3^ McGowan Institute for Regenerative Medicine and Department of Bioengineering, University of Pittsburgh, Pittsburgh, PA, United States; ^4^ Department of Congenital Heart Disease and Cardiovascular Surgery, Fundación CardioInfantil Instituto de Cardiología, Bogotá, Colombia

**Keywords:** anti-thrombotic performance, polyester urethane urea (PEUU), biocompatibility, surface modifications, regenerative cardiovascular devices

## Abstract

**Introduction:** Thrombogenesis, a major cause of implantable cardiovascular device failure, can be addressed through the use of biodegradable polymers modified with anticoagulating moieties. This study introduces a novel polyester urethane urea (PEUU) functionalized with various anti-platelet deposition molecules for enhanced antiplatelet performance in regenerative cardiovascular devices.

**Methods:** PEUU, synthesized from poly-caprolactone, 1,4-diisocyanatobutane, and putrescine, was chemically oxidized to introduce carboxyl groups, creating PEUU-COOH. This polymer was functionalized *in situ* with polyethyleneimine, 4-arm polyethylene glycol, seleno-L-cystine, heparin sodium, and fondaparinux. Functionalization was confirmed using Fourier-transformed infrared spectroscopy and X-ray photoelectron spectroscopy. Bio-compatibility and hemocompatibility were validated through metabolic activity and hemolysis assays. The anti-thrombotic activity was assessed using platelet aggregation, lactate dehydrogenase activation assays, and scanning electron microscopy surface imaging. The whole-blood clotting time quantification assay was employed to evaluate anticoagulation properties.

**Results:** Results demonstrated high biocompatibility and hemocompatibility, with the most potent anti-thrombotic activity observed on pegylated surfaces. However, seleno-L-cystine and fondaparinux exhibited no anti-platelet activity.

**Discussion:** The findings highlight the importance of balancing various factors and addressing challenges associated with different approaches when developing innovative surface modifications for cardiovascular devices.

## 1 Introduction

Various cardiovascular devices, including vascular grafts, stents, valves, and venous catheters, are intended for blood contact. However, thrombogenesis remains a critical factor contributing to the short- and long-term failure of these devices (Rodriguez-Soto et al., 2021; Rodriguez-Soto et al., 2021). For example, recurrent thrombogenesis is the leading cause of patency loss in vascular grafts for hemodialysis access, with primary patency rates of just 41% after 1 year and 28% after 2 years of implantation (Halbert et al., 2020; Halbert et al., 2020). Although secondary patency can be achieved by mechanically removing the thrombus, 29% of cases require a reintervention for replacement (Guidoin et al., 1992; Guidoin et al., 1992).

In healthy blood vessels, a functional endothelium actively serves to prevent thrombogenesis. In contrast, non-endothelialized surfaces facilitate thrombogenesis through protein adsorption, platelet aggregation, complement activation, and thrombin generation. Thrombogenesis not only obstructs the vessel but also is associated with immune system activity, leading to increased foreign body responses and bacterial colonization in specific applications like catheters and vascular grafts for hemodialysis access. In such circumstances, infection rates can reach up to 44% (Ed Rainger et al., 2015; Ed Rainger et al., 2015; Rodriguez-Soto et al., 2021; Rodriguez-Soto et al., 2021).

Historically, efforts to prevent thrombosis on blood-contacting surfaces have centered on systemic anti-thrombotic or anticoagulant drug administration. Furthermore, there is no conclusive evidence on how oral anticoagulation management improves the outcome of patients requiring the use of vascular grafts. Furthermore, there is no clear evidence regarding the benefits of mid-term and long-term anticoagulation in the vascular graft patency outweighing the increased risk of bleeding (Liang et al., 2017; Liang et al., 2017; Schoenrath et al., 2021; Schoenrath et al., 2021). However, developing surfaces with inherent thrombogenesis prevention capabilities can enhance the prognosis of implanted cardiovascular devices. For example, bioprosthetic heart valves that have been modified to exhibit anti-thrombogenic and anti-immunogenic characteristics have demonstrated encouraging outcomes in *in vivo* studies. Such outcomes indicate that inhibiting the formation of thrombi not only enhances hemocompatibility but also bolsters the performance of medical devices. This is achieved by mitigating the risk of extreme immune reactions triggered by hyperactivation, which in turn supports more effective tissue regeneration (Hu et al., 2020; Liang et al., 2023).

Passive and active surface modifications have been proposed to reduce thrombus formation by minimizing protein adsorption (Irvine et al., 2012; Irvine et al., 2012). The interaction between electrostatic and hydrophobic forces influences protein adherence to surfaces, resulting in an increased entropy due to the displacement of water molecules and counter ions from proteins. Specific adsorbed proteins can present binding sites, for platelets and reendothelialization (Tang et al., 2009; Tang et al., 2009; Liu et al., 2013; Liu et al., 2013). Passive surface modifications, such as low-adherent topographies or PEGylation to improve hydrophilicity, can be employed to modify these processes. However, caution is necessary when considering these approaches for cardiovascular devices that require reendothelialization for optimal clinical outcomes (Valencia-Rivero et al., 2019; Valencia-Rivero et al., 2019). Alternatively, active surface modification mainly focuses on surface functionalization with bioactive molecules that target thrombin generation and fibrin formation. For instance, heparin attachment to the surface, as a catalyst for the inactivation of thrombin and factor X, has been proposed (Janairo et al., 2012; Janairo et al., 2012). The PROPATEN^®^ graft, a PTFE vascular graft modified with heparin and approved by the FDA in 2006, has demonstrated a nearly 16% increase in patency compared to its non-coated counterpart. Although direct thrombin inhibitors like hirudin and bivalirudin have been investigated, they have not been widely adopted. Recent research, however, suggests that combining both alternatives can provide a synergistic effect in anti-thrombotic surface modifications (Zhu et al., 2021; Zhu et al., 2021).

In this work, we propose the use of carboxyl group-modified poly (ester urethane) urea (PEUU-COOH) for functionalization with various anti-thrombotic strategies. PEUU exhibits biodegradability and superior mechanical performance, making it a promising biomaterial for cardiovascular tissue engineering applications. It offers the necessary compliance to withstand blood pressure and facilitates mechanotransduction from flow to cells on the surface, which is required for reendothelialization (Fang et al., 2014; Fang et al., 2014). By considering only one bulk material we were able to independently analyze the efficiency of proposed antiaggregating surface treatments.

First, we examined polymer functionalization with a high molecular weight 4-arm PEG (20,000 mW), which could be an outstanding strategy due to its steric inhibition potential and increased hydrophilicity (Pozzi et al., 2014; Pozzi et al., 2014). We then utilized a similar molecule, polyethyleneimine (PEI 400 mW), shown to prolong prothrombin time by blocking thrombin-catalyzed fibrin formation (Chu et al., 2003; Chu et al., 2003). Additionally, considering that heparin is one of the most widely used anti-thrombotic surface modifications, we hypothesized that a synthetic heparin analog, Fondaparinux, might also be suitable for anti-thrombotic functionalization. Fondaparinux selectively inhibits factor Xa and thrombin generation but not its activity, thereby allowing protein C activation for its anticoagulation function. Furthermore, heparin is anti-angiogenic due to the downregulation of endothelial cell migration genes (Shen et al., 2011; Shen et al., 2011). Fondaparinux may offer a selective anticoagulant strategy without interfering with the reendothelialization necessary for regenerative cardiovascular devices (Zhang et al., 2019; Zhang et al., 2019).

Lastly, nitric oxide (NO) released from endothelial cells has been recognized for its antiplatelet function through the inhibition of glycoprotein Ib (GPIb)-V-IX and integrin αIIbβ3 complex in platelets (Yau et al., 2015; Yau et al., 2015). Moreover, NO activates the cGMP (cyclic guanosine monophosphate) dependent protein kinase pathways and reduces cytosolic Ca^2+^, thus inhibiting platelet adhesion, and aggregation, and disrupting platelet aggregates (G.-R. Wang et al., 1998; Wang et al., 1998). Consequently, we hypothesized that surfaces capable of inducing NO production could prevent platelet activation. For this, we selected an organoselenium compound to increase nitric oxide from blood plasma S-nitrosothiols (Yang et al., 2008; Yang et al., 2008; An et al., 2015; An et al., 2015). Furthermore, increased NO levels have been shown to promote endothelial cell proliferation and reduce the inflammatory response of biomaterials by inhibiting proinflammatory cytokine production (Tousoulis et al., 2012; Tousoulis et al., 2012), all of which are desirable properties for regenerative applications.

Building upon these principles, our study aims to develop and evaluate the effectiveness of these anti-thrombotic strategies on the modified PEUU (PEUU-COOH) surfaces. We investigate the biocompatibility, mechanical properties, and anti-thrombotic performance of the functionalized PEUU, comparing each strategy’s efficacy *in vitro* and *in vivo*.

## 2 Materials and methods

### 2.1 Materials

For polymer synthesis, Polycaprolactone diol (PCL, Mn = 2000), dimethylolpropionic acid (DMPA), 1,4-Diisocyanatobutane (BDI), putrescine and stannous octoate (Sn(Oct)2) were purchased from Sigma-Aldrich (St. Louis, MO, USA). 1,1,1,6,6,6-Hexafluoroisopropanol (HFIP) was purchased from Oakwood Products, Inc. (Columbia Hwy, N, Estill, USA). For polymer functionalization, Heparin sodium salt from porcine intestinal mucosa (H3393), Fondaparinux sodium (SML1240), Polyethyleneimine mW 400 (PEI - P3143), 4-arm Polyethylene Glycol with amine terminal groups Mw 20.000 (PEG 4 Arm NH2 - JKA7026), Seleno-L-cystine (545996), N-(3-Dimethylaminopropyl)-N′-ethyl carbodiimide hydrochloride (EDC - E7750), N-Hydroxysuccinimide (NHS - 130672), Chloroform (99.8%) and hydrofluoric acid (48%) were purchased from Sigma-Aldrich (St. Louis, MO, USA). For nanoparticles synthesis used in the functionalization of heparin and fondaporinux surfaces, hydrochloric acid (HCl, 37%, CAS 7647-01-0), glutaraldehyde (GTA, 25%, CAS 111-30-8), acetone (99.5%, CAS 67-64-1), glacial acetic acid (99.7%, CAS 64-19-7), and sodium hydroxide (NaOH, 98%, CAS 1310-73-2) were purchased from PanReac AppliChem (Chica-go, IL, USA). For biological assays, Triton X-100, Phosphate Buffer Saline (PBS), thiazolyl blue tetrazolium bromide (MTT), dimethyl sulfoxide (DMSO, 99%), Dulbecco’s modified Eagle’s medium (DMEM), Roswell Park Memorial Institute 1640 Medium (RPMI1640), and Fetal Bovine Serum (FBS) were purchased from Sig-ma-Aldrich (St. Louis, MO, USA). VERO cells (CCL-81) and THP-1 cells (ATCC TIB-202) were acquired from ATCC^®^. Lactate Dehydrogenase (LDH) kit was acquired from Sigma Aldrich (MAK066, St. Louis, MO, USA). The Nitric Oxide Assay kit was acquired from Abnova (KA1641, Taipei, Taiwan). Fetal bovine serum (FBS) was obtained from Biowest (Riverside, MO, USA), and Type B gelatin was purchased from the local store Químicos Campota (Bogotá, Colombia).

### 2.2 Biomaterial synthesis and modification

#### 2.2.1 PEUU-COOH (PC) synthesis and modification

To facilitate functionalization with various anti-thrombotic bioactive molecules, carboxyl groups were incorporated into the PEUU backbone (Guan et al., 2002; Guan et al., 2002; Hong et al., 2012; Hong et al., 2012; Zhou et al., 2014; Zhou et al., 2014). Briefly, PCL and DMPA were dried in a vacuum oven at 60°C overnight to remove residual water, and BDI and Putrescine were purified through vacuum distillation before PC synthesis. Sn (Oct)_2_ was dried using 4 Å molecular sieves.

**FIGURE 1:**
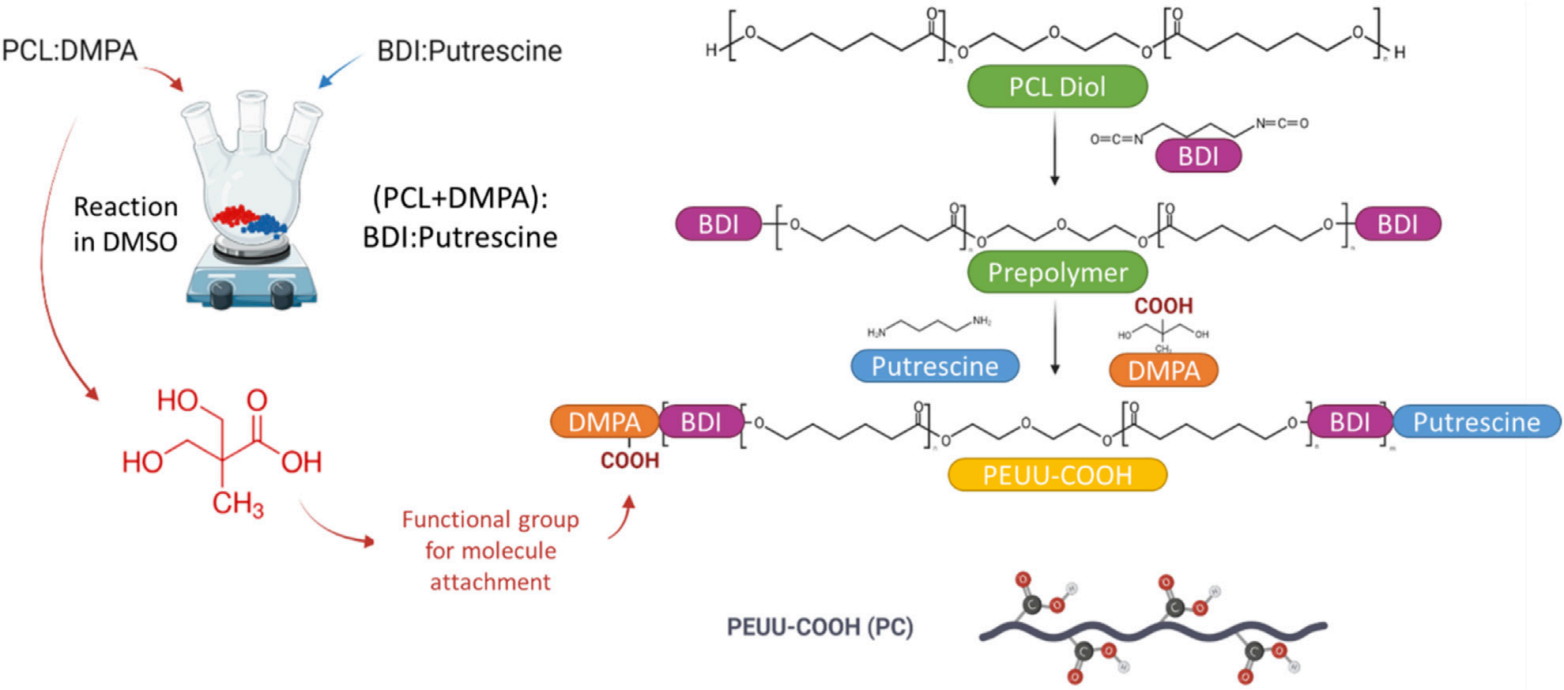
Schematic representation of the workflow for the Synthesis of PEUU-COOH (PC), detailed structure can be seen on Supplementary Figure S1.

PCL was used as the soft segment. DMPA and putrescine are chain extenders and BDI is the hard segment. PCL was dissolved in DMSO in a three-necked flask under argon protection stirring at 75°C. BDI was added followed by 3 drops of Sn (Oct)_2_ as a catalyst. The reaction was allowed for 3 h at 75°C and then the prepolymer solution was cooled at room temperature. DMPA dissolved in DMSO was added dropwise to the pre-polymer solution and the reaction was allowed for 1 h at 25°C. Putrescine diluted in DMSO was slowly added to the reactor then the reactor was kept at 25°C overnight under argon. PCL/DMPA/BDI/putrescine molar ratio was 1:0.5:2:0.5, for a final polymer concentration of 4% on the solution. The polymer was then precipitated with cold deionized water, rinsed with isopropyl alcohol, and dried in a vacuum oven at 60°C for 3 days to obtain fine PC pellets. The reaction yield was over 90%. Figure 1 presents a schematic representation of the PEUU synthesis and carboxylation process. The chemical structure of the synthesized PC was confirmed by proton nuclear magnetic resonance (^1^H-NMR) spectrum and the ratio of PCL:BDI in the backbone was calculated as 1:2.6 from the spectrum (Supplementary Figure S1).

#### 2.2.2 PEUU-COOH (PC) physicochemical characterization

PEUU carboxylation was confirmed through an X-ray photoelectron spectroscopy (XPS), a Fourier-Transform Infrared Spectroscopy (FTIR), and a thermogravimetric analysis.

A photoelectron spectrometer (SPECS Surface Nano Analysis GmbH, Germany) equipped with a PHOIBOS-150 hemispherical electron energy analyzer and a µfocus-600 Al X-ray source was used for XPS measurements. The ultra-high vacuum conditions were kept below 3 × 10^−9^ mbar in the operation procedure. The surface charge compensation of the samples, previously mounted on a non-conductive tape, was achieved by an electron flood gun operated at 3 eV (20 µA) over a tantalum mesh with a nominal aperture of 430 µm. The spot diameter was 200 nm, the energy pass was fixed at 20 eV and the scan number for the high-resolution measurements was 20. The signals were calibrated to a binding energy of 284.6 eV for adventitious carbon and the Ta4f7/2 peak from the tantalum mesh was employed as a reference. The HR spectra were fit by the XPSPeak4.1 software, using a Shirley-type single-peak background with a simultaneous GL peak shape of 30% and full-width at half maximum (FWHM) data from the literature. A deconvolution process was conducted to analyze the surface chemical species after the functionalization of the involved materials.

An Alpha II FTIR Eco-ART (Bruker Optik GmbH, Ettlingen, Germany) was used for the identification of the oxygen-containing functional groups by recording the infrared spectra from 4000 to 600 cm^-1^ with a 2 cm^-1^ spectra resolution. Changes in the characteristic peaks of PEUU were analyzed to identify the backbone structural modifications.

To evaluate alterations in the covalent bonds of the PEUU backbone, a thermal degradation profile was determined using thermogravimetric analysis (TGA) and differential scanning calorimetry (DSC) assays. Both experiments were conducted using a TA Instrument Q600 Thermogravimetric Analyzer^®^ (New Castle, DE, USA). The thermogravimetric profile was obtained under a nitrogen atmosphere (100 mL/min) at a heating rate of 10°C/min, reaching up to 600°C, following ASTM E1131. The DSC analysis was carried out under a nitrogen atmosphere (300 mL/min) at a heating rate of 10°C/min, reaching up to 130°C, and maintained at an isothermal condition for 5 min to eliminate prior thermal history. Subsequently, samples were cooled at a rate of 10°C/min and reheated to 130°C for a second time.

For the COOH quantification, a titration of PEUU and PC was performed. A 30 mL solution of 1 mg/mL of the polymer was resuspended in distilled water, and the pH was adjusted to 3 by adding 0.1 N HCl. Then, 20 μL of 0.1 N, NaOH was added to the solution, and the resulting pH was recorded. A titration curve was built to determine the equivalence point. The content of carboxyl groups was calculated through the product of the titrant concentration (N) and volume at the equivalence point (V), divided by the polymer mass (M) (Barbosa et al., 2013; Barbosa et al., 2013).

#### 2.2.3 PEUU-COOH (PC) anti-thrombotic modification

PC was subsequently functionalized *in situ* with a series of bioactive molecules possessing anti-thrombotic properties. These molecules were divided into two groups for further functionalization. The first group, referred to as “Direct,” encompassed bioactive molecules with NH_2_ functional groups, enabling the formation of a peptidic-covalent bond with the COOH functional groups of PC. The molecules in this group included Polyethyleneimine Mw 400 (PEI), 4-arm Polyethylene Glycol with amine terminal groups Mw 20,000 (P4A), and Seleno-L-cystine (SLC). The second group, designated “Indirect,” involved bioactive molecules containing functional COOH groups, necessitating the use of a linker. Gelatin nanoparticles with NH_2_ functional groups were employed to establish a bond between PC and the anticoagulant molecule. This group comprised molecules such as heparin sodium salt derived from porcine intestinal mucosa (HEP) and fondaparinux sodium (FPX).

For direct *in-situ* functionalization, PC pellets were dissolved in chloroform (5% w/v) at 30°C under magnetic stirring. Then, according to the number of COOH functional groups quantified in the PC (3 × 10^−4^ mol/g), a 1:10 ratio of polymer/EDC and NHS were added (3 × 10^−3^ mol/g), and the activation reaction was allowed for 15 min at 37°C under magnetic stirring. Following this, the anti-thrombotic bioactive molecule with NH_2_ functional groups was added to the solution and the reaction was allowed for 24 h at 50°C under magnetic stirring. In this regard, a 1:2 ratio was considered for PEI (2 × 10^−4^ mol/g) and P4A (2 × 10^−4^ mol/g), while a 1:1 ratio was considered for SLC (2 × 10^−4^ mol/g). Upon completion of each reaction, solutions underwent solvent casting for 24 h, and the resulting film was washed twice with Type II water.

For indirect *in-situ* functionalization, the gelatin B nanoparticles (GNPs) were synthesized using a previously reported two-step desolvation method (Gonzalez-Melo et al., 2021; Gonzalez-Melo et al., 2021). Briefly, Type B gelatin was dissolved in type II water (5% w/v) and an equal volume of acetone was added dropwise for 5 min to induce coiling. The supernatant was collected and centrifuged at 810 *g* for 3 min to recover the high molecular weight gelatin. The resulting pellet was resuspended in an equal volume mixture of water and acetone, and the pH was adjusted from 10.5 to 11.5 before initiating the second desolvation process. Chemical crosslinking of the NH_2_ groups to form the GNPs was achieved by gradually adding glutaraldehyde to a final concentration of 0.4% w/v while stirring magnetically and allowing the reaction to proceed for 16 h. Excess acetone was removed by evaporation, with simultaneous water addition to prevent agglomeration, and the GNPs were recovered by freeze-drying.

**FIGURE 2:**
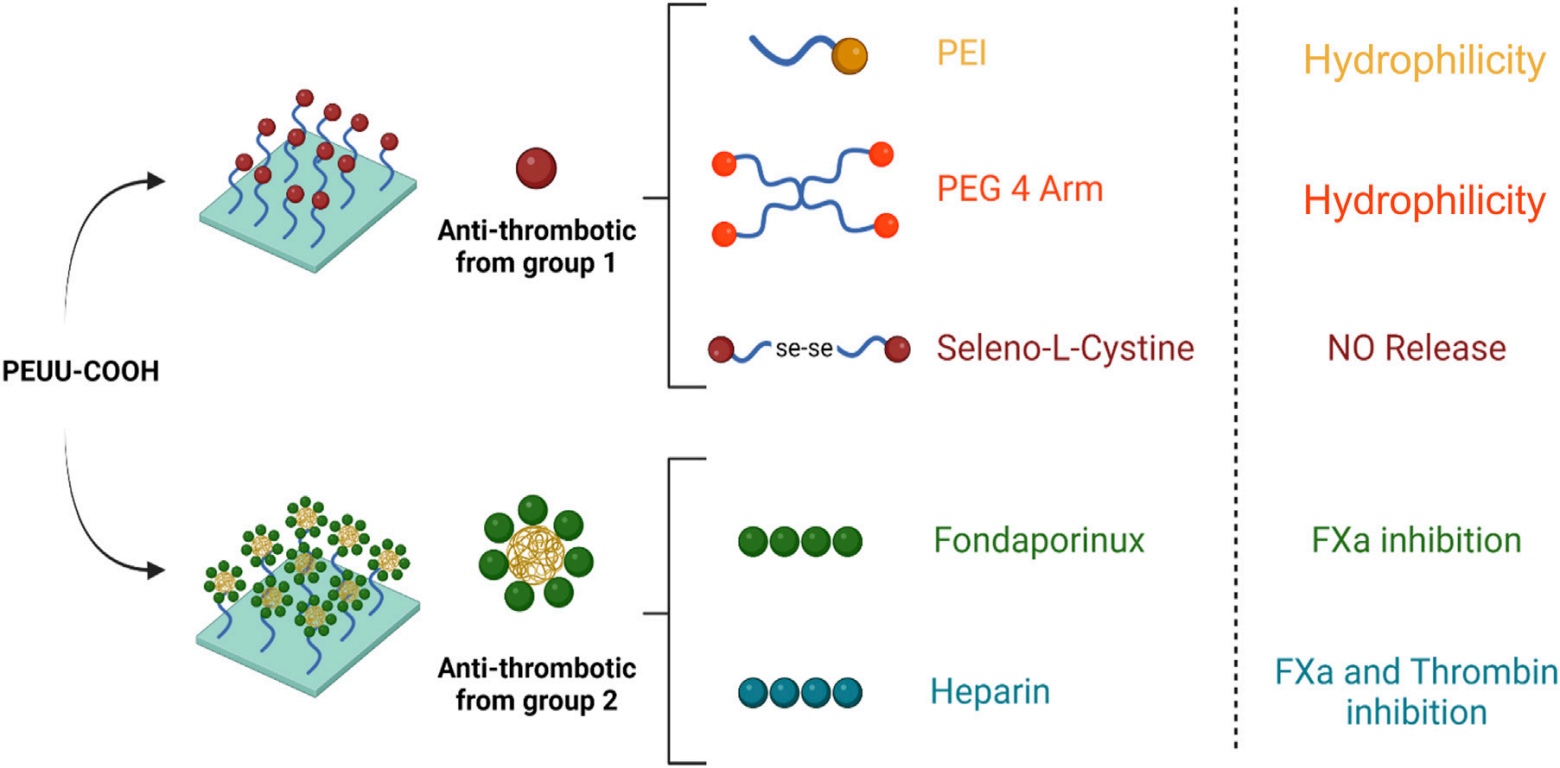
Schematic representation of the PEUU-COOH (PC) Functionalization. Group 1–Direct Functionalization. Group 2 –Indirect Functionalization.

For indirect functionalization, PC pellets were dissolved in chloroform (2.5% w/v) at 30°C under magnetic stirring. Then, a 1:10 ratio of polymer/EDC and NHS (3 × 10^−3^ mol/g) was added, and the activation reaction proceeded for 15 min at 37°C under magnetic stirring. Subsequently, a 1:10 ratio of polymer/GNPs (3 × 10^−3^ mol/g) and a 1:5 ratio of HEP or FPX (2 × 10^−3^ mol/g) were added. The mixture was allowed to react for 24 h at 50°C under magnetic stirring. Each solution underwent solvent casting for 24 h and the obtained film was washed twice with Type II water. Figure 2 Shows a schematic representation of the PEUU-COOH (PC) Functionalization. Two different groups were assigned as Group 1–Direct Functionalization and Group 2–Indirect Functionalization.

To confirm surface functionalization, XPS and FTIR spectra were obtained and analyzed following the previously described procedure. The primary peaks of the raw bioactive molecules were compared with the functionalized surfaces on the PC. TGA and DSC assays were also performed as previously described to assess differences in thermal degradation profiles resulting from the chemical modification of the PC films. Films, each measuring 1 cm^2^ films were sterilized with ethylene oxide at the sterilization center of Fundación CardioInfantil following the NTC 4426-1 and NTC 4426–2 standards, ahead of the subsequent biocompatibility and anti-thrombogenic tests. Surface hydrophilicity was evaluated by measuring the contact angle using a 100 μL droplet of Type II water before and after sterilization.

To elucidate the nitric oxide production capacity of PC functionalized with seleno-L-cystine (PC + SLC) and its relation with anti-thrombotic activity, we quantified the total NO_2-_/NO_3-_ released by plasma in contact with the functionalized polymer as an indicator of NO production. Briefly, O+ fresh human blood was collected in sodium citrate blood collection tubes after obtaining informed consent (Ethical Committee at the Universidad de Los Andes, minute number 928-2018). The anticoagulated blood was centrifuged at 324 *g* for 15 min to obtain platelet-poor plasma (PPP). Films were then exposed to 400 μL of PPP, followed by incubation for 24 h at 37°C and 5%CO_2_. The supernatant was collected, deproteinized with ZnSO_4_ and NaOH according to the manufacturer’s instructions (KA1641, Taipei, Taiwan), and centrifuged at 2250 *g* for 10 min to remove the protein pellet. The reaction proceeded via the reduction of nitrates to nitrite using the Griess method, incubating the sample with the required A and B reagents for 10 min at 60°C. Centrifugation was performed at 2250 g for 30 s, and the supernatant was transferred to a brand new 96 well-plate to measure absorbance at 540 nm. Controls included pristine PC and PPP alone. NO concentration was calculated using a linear dependency of y = 0.003x + 0.07 from a linear regression model according to nitrite standard curve with a known nitrite concentration (100 uM), and NO release was then normalized based on the surface area.

### 2.3 Biocompatibility analysis

Considering that the films presented in this study are intended to be in contact with live tissues and blood, cytocompatibility, and hemolytic propensity were assessed according to the ISO 10993 standard. For the hemolysis test, O+ fresh human blood was collected in Ethylenediaminetetraacetic acid (EDTA) blood collection tubes after obtaining informed consent (Ethical Committee at the Universidad de Los Andes, min number 928-2018). To isolate erythrocytes and remove the plasma contents, the anticoagulated blood was washed five times with a 0.9% w/v NaCl physiological solution at 324 g for 5 min. The isolated erythrocytes were resuspended in PBS 1x to obtain an initial stock of 4 × 10^6^ erythrocytes/μL. Sterilized films of 0.3 cm^2^ were immersed in 150 μL of the erythrocyte solution, while 1x PBS and 1% v/v Triton X-100 served as negative and positive controls, respectively. The samples were incubated at 37°C for 1 h, centrifuged, and the supernatant absorbance was determined at 454 nm. The hemolysis percentage was determined by means of the positive control with Triton X-100 (International Organization for Standardization, 2017; International Organization for Standardization, 2017).

To evaluate cytocompatibility, a metabolic activity assay was performed using MTT on VERO cells and THP-1 cells. For this purpose, 1.0 × 10^5^ VERO cells/mL were seeded onto 96-well plates containing DMEM supplemented with 10% FBS and allowed to adhere for 24 h at 37°C in a 5% CO_2_ atmosphere. To assess cytocompatibility, the MTT metabolic activity assay was performed on VERO cells and THP-1 cells. VERO cells (1.0 × 105 cells/mL) were seeded on 96-well plates with DMEM supplemented with 10% FBS and allowed 24 h for adhesion at 37°C in a 5% CO2 atmosphere. In parallel, THP-1 cells (5.0 × 105 cells/mL) were seeded on 96-well plates containing RPMI 1640 supplemented with 10% FBS. Cells were then exposed to previously sterilized 0.4 cm^2^ films for an additional 24 h and 72 h in serum-free media. DMSO at a concentration of 10% v/v and untreated cells were used as negative and positive controls, respectively. Post incubation, films were carefully removed, and the MTT solution was applied to facilitate formazan crystal formation over a 2-h incubation period. Media was removed after centrifuging the culture plates at 250 g for 5 min, and DMSO was added to dissolve the formazan crystals. Absorbance was then read at 595 nm, with cell viability percentages benchmarked against a live cell control (International Organization for Standardization, 2017; International Organization for Standardization, 2017).

### 2.4 Anti-thrombotic activity analysis

To assess the anti-thrombotic activity of the different bioactive molecules employed, multiple assays were performed to examine protein adsorption, platelet aggregation, activation, and clot formation.

We hypothesized that surface modifications increasing wettability would also reduce platelet aggregation due to potential alterations in the protein adsorption profile. Therefore, the contact angle was determined by directly measuring the tangent angle at the interfacial point when a 100 μL drop of type II water reached the three-phase equilibrium point on a 1 cm^2^ sample before and after sterilization. To evaluate the protein adsorption capacity, 0.5 cm^2^ samples were immersed in 10% FBS and incubated for 12 h. The samples were then transferred to a 96-well plate and washed with 50 μL of a 1% SDS solution for 35 min at 37°C under gentle agitation at 100 rpm. Supernatant protein concentrations were quantified using a Bicinchoninic Acid (BCA) Assay Kit (Quanti-Pro, Sigma Aldrich). Solutions with and without FBS in the absence of any treatment served as positive and negative controls, respectively. BCA working solution incubation was allowed for 30 min and absorbances were measured at 565 nm. Protein concentration was calculated using a linear dependency of y = 0.2633x + 0.1019 from a linear regression model according to a Bovine serum albumin (BSA) standard curve with a known protein concentration (2 mg/mL), and protein concentration was then normalized based on the surface area.

For platelet aggregation and activation, O+ fresh human blood was collected in sodium citrate blood collection tubes after obtaining informed consent (Ethical Committee at the Universidad de Los Andes, min number 928-2018). The anticoagulated blood was centrifuged at 180 *g* for 10 min to obtain platelet-rich plasma (PRP). Films of 0.5 cm^2^ films were then exposed to 200 μL of PRP that had been activated with 20 μL 0.1 M CaCl, allowing contact for 20 min. After incubation, films were removed and the supernatant absorbance was determined at 620 nm. Surfaces functionalized with epinephrine, adenosine diphosphate (ADP) and collagen were used as positive controls with high, medium, and low aggregation potential, respectively. Platelet aggregation was expressed as a percentage of the epinephrine control (International Organization for Standardization, 2017; International Organization for Standardization, 2017).

Scanning electron microscopy (SEM) was used to verify platelet presence and activation. Sterilized 0.5 cm^2^ films were exposed to PRP for 30 min under gentle agitation at 10 rpm. The films were then removed, fixed with glutaraldehyde 4% v/v for 30 min, and washed 3 times with PBS 1x. The samples were dried using a decreasing ethanol curve, affixed onto aluminum plates with carbon tape, and coated with a gold layer using a Vacuum Desk IV apparatus (Denton Vacuum, Moorestown, NJ, USA). An SEM model JSM-6490LV^®^ (JEOL USA Inc., Peabody, MA, USA) with a 10 kV accelerating voltage was used for sample analysis.

An LDH assay was performed to quantify the platelet number adhered to the film surfaces exposed to PRP for 1 h. For quantification, a calibration curve was built using PRP with 10 serial dilutions of 3.56 × 10^5^ platelets/µL, and absorbance was recorded at 493 nm. The exposed films were transferred to brand new plates, and platelets were lysed with 1% Triton X-100 for 5 min. The films were then removed and the LDH working solution (MAK066, St. Louis, MO, USA) was applied to the supernatant with absorbance recorded at 493 nm. The number of platelets was determined with the aid of a linear dependency of y = 4 × 10^−8^ x + 0.1427 obtained from a linear regression model of a standard curve created with decreasing concentrations of PRP. Data were normalized based on the contact surface area (Braune et al., 2015; Braune et al., 2015).

To assess whole blood clotting on the film surfaces, the O+ whole blood from human donors was collected in sodium citrate tubes with the first tube discarded to avoid contamination with tissue thromboplastin. 5 mL of whole blood was transferred to a centrifuge tube, and 500 μL of 0.1 M CaCl was added immediately before the assay to restore coagulability. Sterilized 1.5 cm^2^ films were placed in 12-well plates and 200 μL of the activated blood was applied to the film surface. The samples were incubated for 15 min or 1 h at 37°C to facilitate clot formation. After incubation, 3 mL of type II water was carefully added to each sample and incubated for 5 min. 100 μL of the supernatant was transferred to a 96-well plate and absorbance was recorded at 540 nm. Positive control for coagulation was obtained with a glass slide. Absorbance is proportional to free hemoglobin released by the red blood cells that are not protected by the polymerized fibrin mesh and is inversely related to thrombus formation (Sabino and Popat, 2020; Sabino and Popat, 2020).

### 2.5 Statistical analysis

**FIGURE 3:**
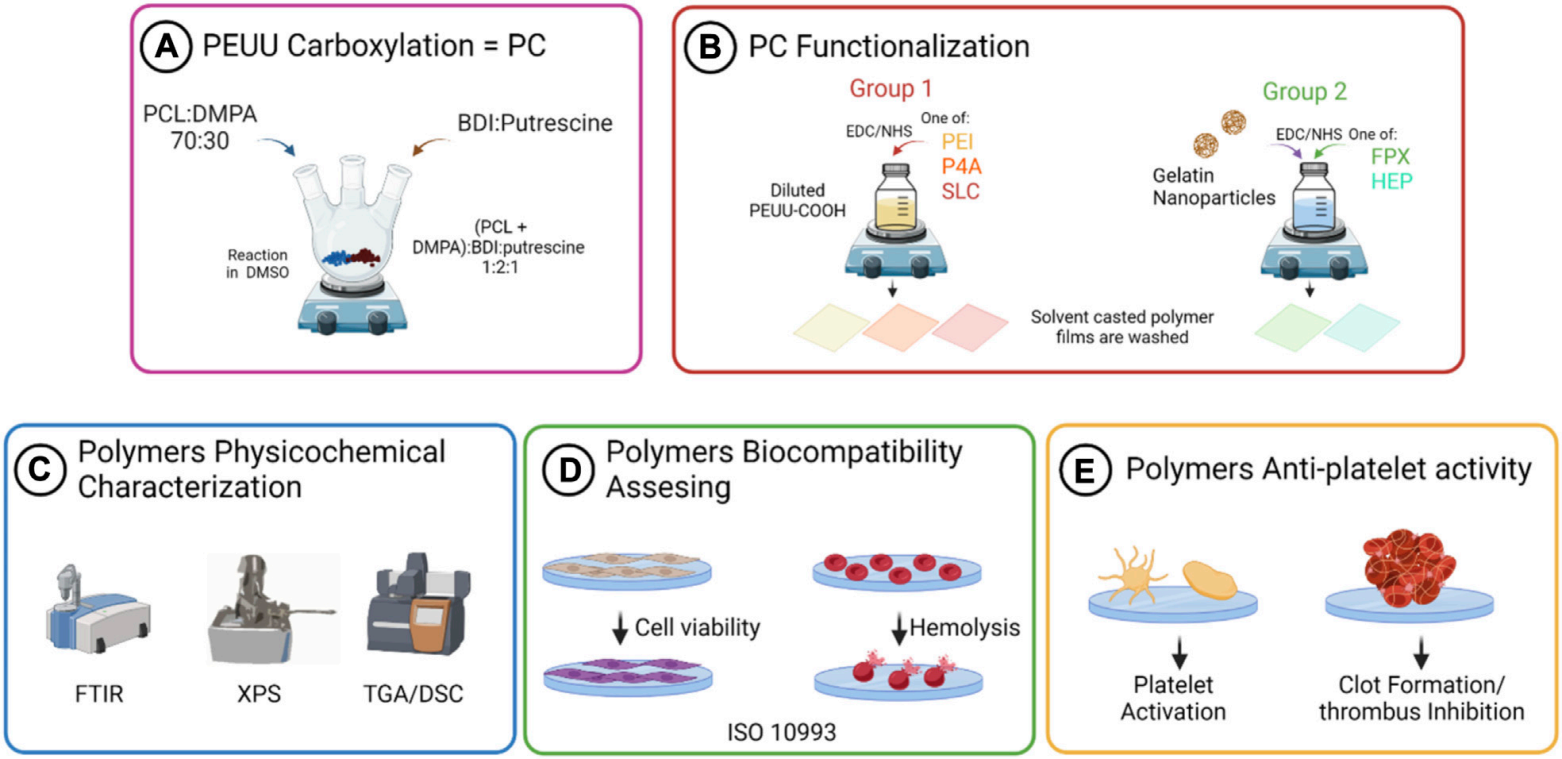
Schematic representation of the workflow for the PEUU-COOH (PC) *in-situ* functionalization with anticoagulant molecules and anti-thrombotic activity assessment. **(A)** PEUU carboxylation with DMPA **(B)** Physicochemical characterization of PEUU-COOH. **(C)** *In-situ* functionalization with anti-thrombotic bioactive molecules. **(D)** Biocompatibility assessment. **(E)** Anti-thrombotic activity assessment.

Statistical analysis of the data was conducted using GraphPad Prism^®^ 9.1.1 software (Windows, GraphPad Software, San Diego, CA, USA, www.graphpad.com, accessed on 24 March 2023). A two-way ANOVA test with Tukey’s multiple comparisons of means was employed after confirming the normality, independence of observations, and homoscedasticity of the data. Data conforming to normal distribution are presented as mean ± standard deviation, with *p*-values less than 0.05 (*p* < 0.05) deemed significant. Grubbs’ test was applied to identify single outliers within the data sets (data not shown). A schematic illustration of the scaffold fabrication process is provided in Figure 3.

## 3 Results and discussion

### 3.1 PEUU carboxylation

#### 3.1.1 PEUU-COOH (PC) X-ray photoelectron spectroscopy (XPS) analysis

**FIGURE 4:**
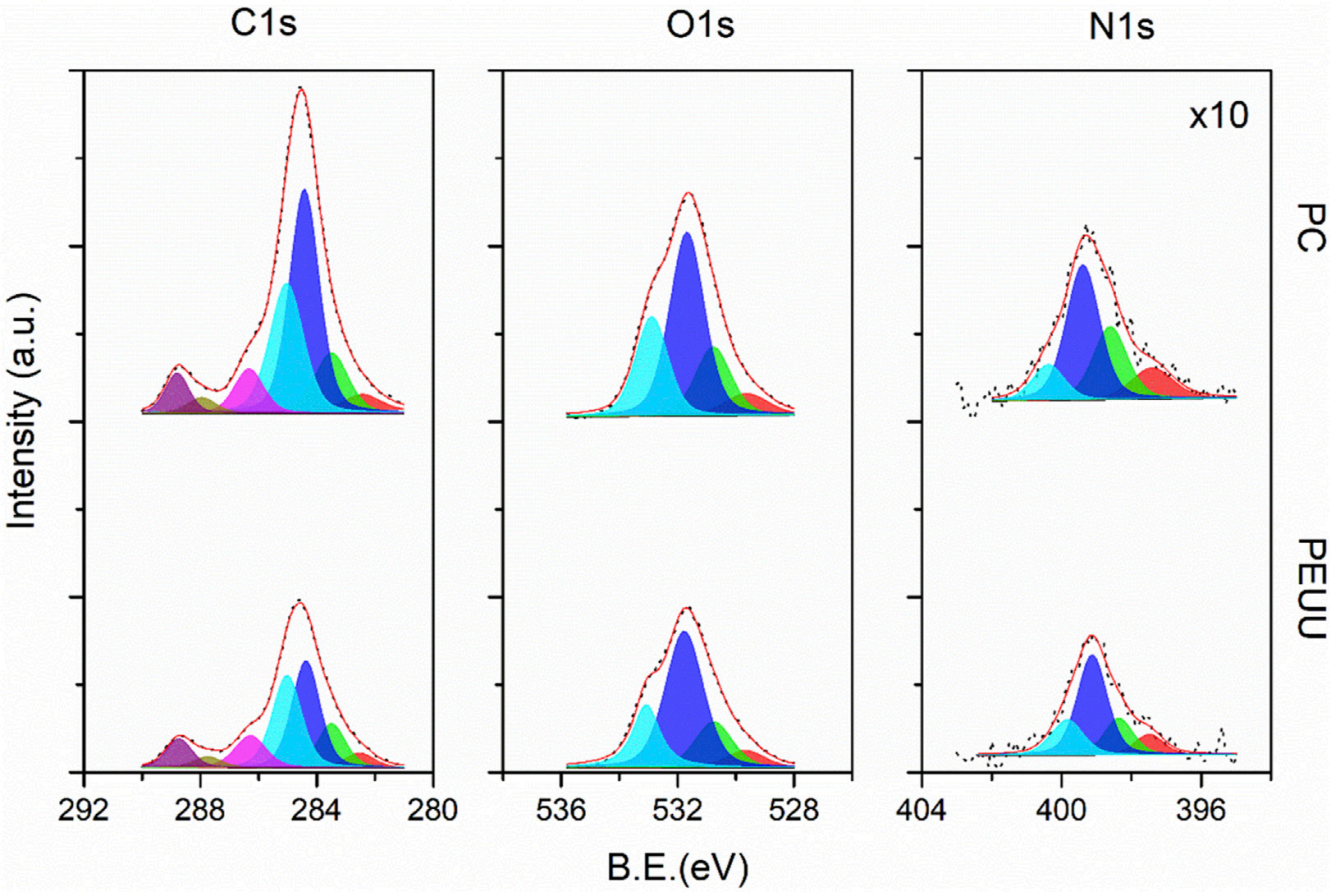
High-resolution XPS spectra for the C1s, O1s, and N1s core-level of PEUU (bottom) and PC (top) samples. Colored areas correspond to the sub-peak components referred to in Supplementary Table S1.

The chemical surface characterization of PC was conducted by the XPS. A reference sample of PEUU reference was analyzed to determine the incorporation of dimethylolpropionic acid (DMPA) into the PEUU backbone. Figure 4 shows the high-resolution (HR) spectra for the main peaks of C1s, O1s, and N1s, subdivided into component sub-peaks delineated by distinct color zones. The spectra were normalized to the C1s mean signal. The binding energy (BE) values for all components, integral to the overall fitting, are indicated by red lines overlaid on black dots that represent the experimentally recorded data A summary of the BE, full width at half maximum (FWHM), and area under the curve values for all analyzed systems is presented in Supplementary Table S1 (Supplementary Section S1). Charging artifacts, evidenced as asymmetric tails at lower energies, are marked in red.

For the C1s core level, both samples showed sub-peaks corresponding to -O-C=O (C6—magenta-), N-C=O (C5—brown), -C-O (C4 –purple), -C-N (C3 –cyan), C-C (C2 –blue) and C-H (C1 –green) bonds, arranged from high to low binding energies. The peak integration counts, calculated from the area under the peak, demonstrated a noticeable disparity; the PC sample had higher counts than the PEUU, attributed to the presence of DMPA molecules. This variation confirms successful functionalization, as the urea/urethane ratio decreased from 1/1 to 1/3 after substituting DMPA for putrescine on a 1:1 basis. This substitution suggests an increased concentration of pi-bond resonant structures, caused by the interaction between DMPA, BDI, and PCL molecules. Consequently, the chemical potential of the PC sample surface exceeded that of PEUU. This observation correlates with the homogeneous distribution of the electronic density of states along the chain extension. It is noteworthy that XPS analysis was conducted on large polymer compounds, thus the fitting data reflect the aggregate chemical environment, directly linked to the functional groups introduced during the carboxylation process.

For the O1s core level, the spectrum consisted of three sub-peak components corresponding to chemisorbed hydroxyl (O3—cyan), -O-C (O2—blue), and C=O (O2—green) bonds, arranged from high to low binding energies. The results align with those of the carbon species since the O1s intensities for the PC sample exceeded those of the reference sample. Similarly, the N1s core-level exhibited weak signals for -N-C=O (N3—cyan), -N-H2 (N2—blue), and --N-H (N1– green) bonds, which are congruent with the organic matrix. Despite the general increase in count observed with the introduction of DMPA molecules, the -N-C=O bond component remained relatively consistent due to the expected reduction of amides with putrescine substitution.

#### 3.1.2 PEUU-COOH (PC) FTIR analysis

**FIGURE 5:**
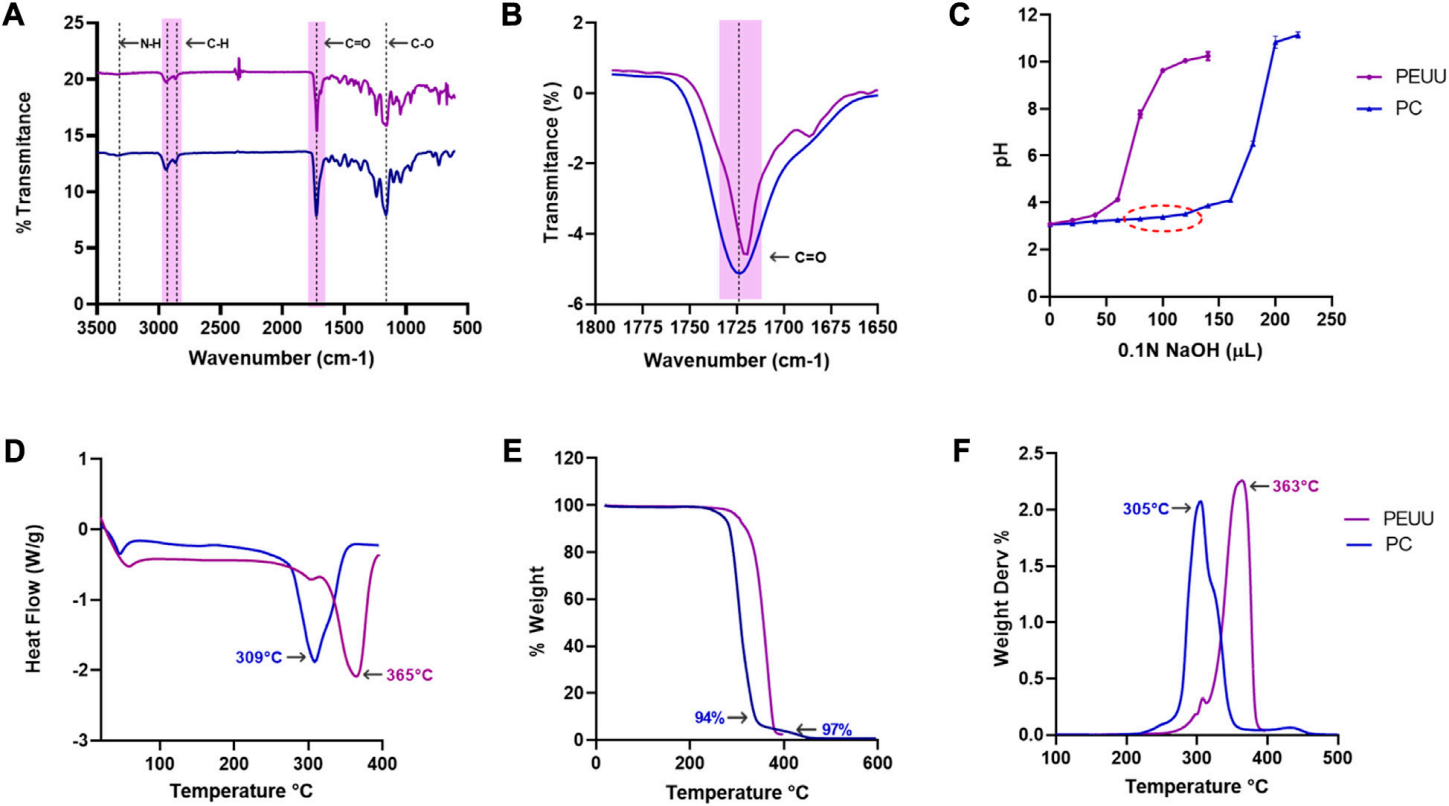
Physicochemical evaluation of the carboxylation of PEUU. **(A)** FTIR spectra of PEUU and PC measured in the range between 3500 and 500 cm^-1^. Magnification of the **(B)** carbonyl (C=O) stretching. **(C)** Titration curve for COOH quantification (Mean ± SD). **(D)** DSC thermograms of PEUU and PC **(E)** Thermogravimetric (TGA) analysis and **(F)** DTGA curves of PEUU and PC.

The physicochemical evaluation of PEUU was conducted by Fourier Transform analysis, which confirmed the presence of functional groups in the polymer and the successful introduction of carboxyl groups of the PEUU (PC). The characteristic peaks of PEUU were identified at 3320 cm^-1^ (urethane and urea N-H stretching vibration), 2933 and 2855 cm^-1^ (carboxyl C-H stretching vibration), 1724 cm^-1^ (ester carbonyl stretching vibration of the urethane group C=O) and 1160 cm^-1^ (stretching vibration of the ester group C-O-C) (Nair and Ramesh, 2013; Nair and Ramesh, 2013; Włoch et al., 2018; Włoch et al., 2018). As shown above (Figures 5A, B) the carbonyl group peak of the PC (1724 cm^-1^) exhibited elongation and broadening, indicating the oxidation of the PEUU and the presence of the COOH free radicals, which confirmed the successful functionalization of the polymer.

Additionally, the titration curve was used to confirm the carboxylation process. The presence of a higher number of carboxyl groups in the polymer results in increased acidity, requiring a greater quantity of NaOH solution to raise the pH level, as shown in Figure 5C. This technique has been widely used to determine the quantification of carboxyl groups in materials, considering the material’s acidity (El Jundi et al., 2020; El Jundi et al., 2020; Babaev et al., 2022; Babaev et al., 2022). Therefore, the results of the titration curve provide additional evidence of the successful carboxylation of PEUU.

#### 3.1.3 PEUU-COOH (PC) thermogravimetric analysis

The weight degradation profile of both PEUU and PC was analyzed via TGA, and the results are presented in Figures 5D, E. The two polymers exhibit similar behavior, but PC displays a distinct degradation temperature compared to PEUU, with temperature peaks dropping from 365°C to 305°C. This variation may be due to structural modifications in the PEUU, leading to weaker bonds or less stability in the carboxylated polymer itself. Alternatively, the decrease in temperature could be due to the reduction in molecular weight when compared to the unmodified polymer (Hong et al., 2012; Hong et al., 2012). Nevertheless, this difference in degradation temperature does not represent a significant inconvenience, as the melting temperature does not differ greatly from that of PEUU. Furthermore, a second weight loss can be observed at 350°C which may be related to the decomposition of urethane and urea bonds, and a total and remaining decomposition at 442 °C related to the decomposition of ester bonds (El-Raheem et al., 2021; El-Raheem et al., 2021).

The DSC thermogram confirmed the TGA results, showing a melting temperature of 363°C for PEUU and 305°C for PC (Figure 5F). The differences in both degradation temperatures can be attributed to the loss of bonds resulting from COOH generation on the backbone of the PC, leading to a slightly less stable polymer. However, these structural changes do not represent critical modifications in the material but are made to facilitate the formation of peptide bonds with the available amine (-NH) terminals of anti-thrombotic molecules studied (Fang et al., 2014; Fang et al., 2014).

### 3.2 PEUU-COOH (PC) biocompatibility and hemocompatibility

As the proposed biomaterial is intended for implantation and will come into contact with blood, its biocompatibility was assessed by evaluating cell viability, hemolysis percentage, and platelet activity.

**FIGURE 6:**
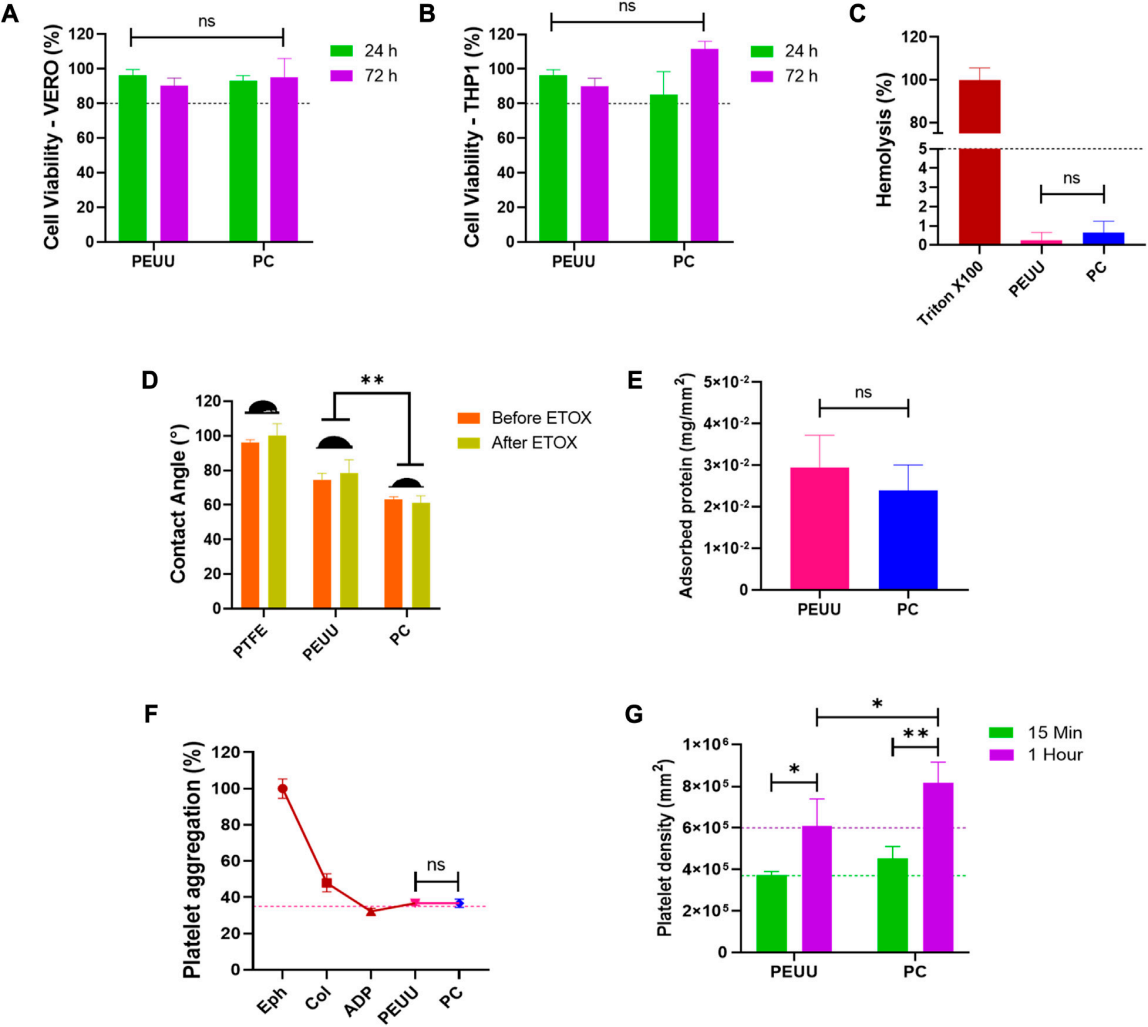
Biocompatibility testing of PC. Viability percentage extracted from MTT data of tests performed by directly exposing **(A)** VERO cell cultures (ATCC CCL81.4) and **(B)** THP-1 Cell cultures (ATCC TIB-202) to film’s fragments for 24 h and 72 h. **(C)** Hemolysis percentage of films exposed to only erythrocytes. **(D)** Contact Angle of type II H2O on the surface of the polymers before and after sterilization with ethylene oxide. **(E)** Adsorbed serum protein on the film surface. **(F)** Platelet aggregation percentage of PRP exposed to films. **(G)**) Number of platelets adhered to the surface of the film by LDH activation analysis. Data compared to PEUU. Mean ± SD) where, ns = no significant *p ≤ 0.05 **p ≤ 0.01 ***p ≤ 0.001 ****p ≤ 0.0001.

Cell viability of VERO cells exposed to the PC film was analyzed at 24 and 72 h (Figure 6A). Results indicated no significant decrease in viability for both polymers, with PC showing cell viability percentages of 93% ± 11 at 24 h and 95% ± 11 at 72 h, compared to PEUU at 96% ± 4% and 90% ± 4, respectively. No apparent cell morphological changes were observed, and both maintained a viability percentage above the minimum allowed by the 10993 ISO standard (80%). Similar results are found when the viability of exposed THP-1 cells was evaluated (Figure 6B). PEUU-COOH showed 87% ± 4 and 111 ± 4 after 24 h and 72 h, while PEUU maintained the viability at 96% ± 5% and 91% ± 5 after 24 h and 72 h. No statistically significant differences were found.

The hemolysis percentage (Figure 6C) of the PC remained below 1% (0.6% ± 0.6) similar to PEUU (0.2% ± 0.4), with no statistically significant differences, and well below the maximum percentage allowed by the 10993 standards (5%), indicating hemocompatibility for both polymers.

To further analyze anti-thrombotic activity, contact angle (Figure 6D) and protein adsorption (Figure 6E) profiles were examined. Findings revealed that polymer functionalization led to a decrease in contact angle and an increase in hydrophilicity, this behavior did not present any significant difference after ethylene oxide sterilization. Post-sterilization contact angle measurements yielded 64 ± 6 for PEUU-COOH *versus* 78 ± 5 for PEUU (*p* ≤ 0.01), with a PTFE graft serving as a hydrophobic control (96 ± 2). No discernible differences were observed post-sterilization in either film’s manipulability or flexibility. Previous reports have shown that ethylene oxide sterilization might cause an increase on the rigidity of the biomaterial, thus mechanical property assessment is crucial for each specific application (Bednarz et al., 2018).

PC had lower mean protein adsorption than PEUU (3 × 10^−2^ mg/mm^2^ ± 8 × 10^−3^ vs. 2 × 10^−2^ mg/mm^2^ ± 6 × 10^−3^), though these surfaces were not statistically different. According to Ramachandran, B. et al., PEUU’s wettability can be influenced by both the density of carboxyl groups present on its surface and its surface roughness. Consequently, PC exhibits more pronounced hydrophilic behavior in comparison to the unmodified polymer. As the carboxyl group density on the polymer surface increased, protein adsorption decreased (Ramachandran et al., 2018; Ramachandran et al., 2018).

Upon 20 min of exposure to PRP, the platelet aggregation rate elicited by the PEUU-COOH was comparable to that of PEUU, registering 37% ± 1 *versus* 36% ± 2 (*p* ≤ 0.05) (Figure 6E). Films functionalized with epinephrine, collagen, and ADP served as platelet aggregator inducers, categorizing them as high (100% ± 5), medium (47% ± 5), and mild (32% ± 1) aggregators, respectively. To determine if this result was related to platelet activation, an LDH test was performed, and the total count of adhered platelets/mm^2^ was calculated through a linear regression from a calibration curve (Figure 6). Findings showed that the number of platelets adhered to the film surface increased proportionally with time, with a statistically significant increase from 15 min to 1 h for both groups. The increase in platelets adhered to surfaces was from 4 × 10^5^ ± 2 × 10^4^/mm^2^ at 15 min to 6 × 10^5^ ± 1 × 10^5^/mm^2^ at 1 h for PEUU (*p* ≤ 0.05), and from 3 × 10^5^ ± 2 × 10^5^/mm^2^ at 15 min to 8 × 10^5^ ± 1 × 10^5^/mm^2^ at 1 h for PC (*p* ≤ 0.01). Although platelet aggregation was lower for PC than for PEUU, platelet adhesion to the modified surface was twice as high, and the platelet density increased at a faster rate over time. This may be attributed to the presence of -COOH free radicals, which facilitate platelet adhesion, a property that PEUU does not possess. Platelet aggregation is primarily driven by platelet-to-platelet adhesion, a process initiated by an elevation in the intracellular calcium levels. This increase subsequently boosts the activity of the GPIIb/IIIa complex, which serves as an adhesive molecule. Given this mechanism, the carboxyl groups present on PC could potentially serve as anchoring sites for platelet adhesion (Poussard et al., 2004). Furthermore, previous research has highlighted that the negative charge provided by the terminal carboxyl groups has the capacity to activate the FXII coagulation factor, further promoting platelet adhesion (Stavrou and Schmaier, 2010; Shiu et al., 2014). Importantly, cell viability can be influenced by a myriad of factors, among which surface charge and the types of functional groups on the surface stand out as particularly significant.

### 3.3 PEUU anti-thrombogenic functionalization

Surface functionalization was classified into two distinct groups based on the strategy employed for incorporating anti-thrombotic molecules. Group 1 involved direct functionalization, while Group 2 necessitated indirect functionalization using gelatin nanoparticles as intermediaries between the polymer and the bioactive molecules.

In Group 1, anti-thrombotic molecules were directly grafted onto the polymer surface, facilitating a straightforward interaction between the polymer and the molecules. This approach aimed to enhance the inherent anti-thrombotic properties of the modified polyester urethane urea (PEUU) surface, potentially improving the performance of regenerative cardiovascular devices. Conversely, Group 2 employed gelatin nanoparticles as a bridge between the polymer and the bioactive molecules. This indirect functionalization strategy allowed for the controlled release of anti-thrombotic agents and facilitated a more stable interaction between the polymer and the bioactive molecules. The utilization of gelatin nanoparticles as a delivery system may offer additional benefits, such as improved biocompatibility and the ability to incorporate multiple bioactive agents for synergistic effects.

#### 3.3.1 PEUU-COOH (PC)/anti-thrombogenic functionalization XPS analysis

**FIGURE 7:**
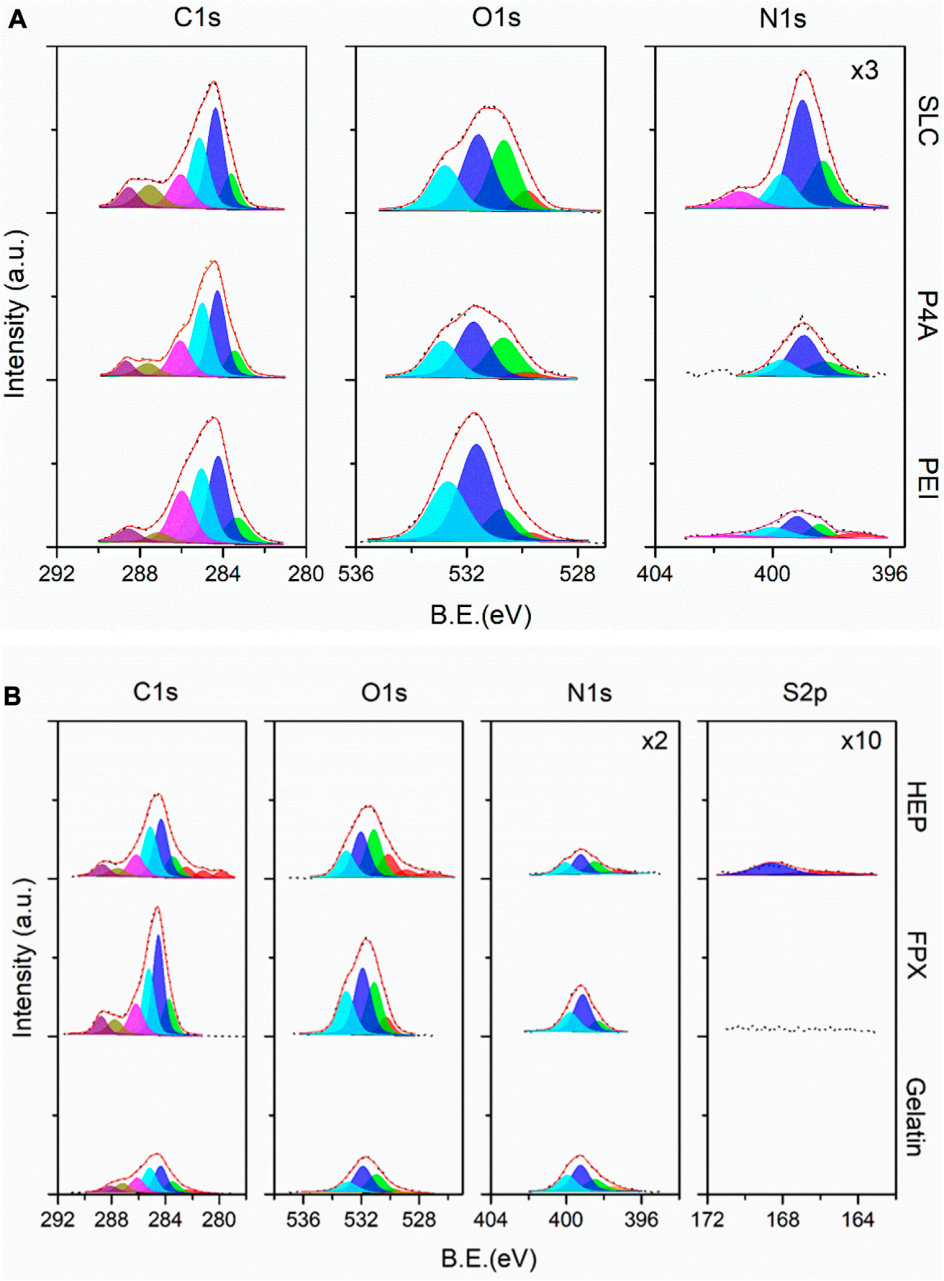
**(A)** High-resolution XPS spectra for SLC, P4A, and PEI samples (top-down) corresponding to the Group 1 of the PEUU-COOH (PC)/Anti-thrombotic functionalization system. **(B)** High-resolution XPS spectra for HEP, FPX, and gelatin (reference) samples (top-down) corresponding to the Group 2 of the PEUU-COOH (PC)/Anti-thrombotic functionalization system.

The surface chemistry evaluation of the functionalized samples was organized into two categories. The first involved a direct reaction of Seleno-L-Cystine (PC + SLC), PEG 4 ARM (PC + P4A), and polyethyleneimine (PC + PEI). The high-resolution XPS spectra for these reactions are displayed in Figure 7A (top-down). The results and discussion are drawn from Section 3.3.1, and henceforth, all three systems will be discussed concurrently for each core level. Beginning with the C1s peak, atomic percentages linked to carboxyl and amide functional groups escalated in a sequence from PEI, P4A, to SLC samples. Contrarily, the C-O interaction was more pronounced in the PEI sample, while hydroxyl species peaked as anticipated. These observations correlate well with the N1s core level, where the peak intensity also ascended in the same sequence. Protonated species were readily discernible at a binding energy of 401.1 eV (N4—blue). In addition, carbonyl species were scrutinized. The O1s subpeak suggested a superior functionalization yield for the SLC sample, trailed by P4A and PEI, in that order.

The second part of the evaluation involved an indirect, *in-situ* functionalization, as depicted in the high-resolution XPS spectra presented in Figure 7B. This section includes gelatin nanoparticles (GNPs) as a reference, with Fondaparinux (PC + FPX) and Heparin (PC + HEP) serving as anti-thrombogenic agents. Significant changes were observed in the overall intensity of the anti-thrombogenic agents relative to the GNPs. Moreover, charge artifacts were more prominent for the PC + HEP sample. This finding suggests a successful surface modification, given the constant electron bombardment maintained throughout the experimental setup across different sample types. To delve further, the S2p core level was analyzed, confirming the presence of sulfate species at 168 eV for PC + HEP. This was not observed for PC + FPX. However, both samples demonstrated an increased count of -C=O bond types and a considerably higher atomic percentage of oxygen species compared to GNPs. The analysis of the N1s core level revealed that GNPs were fully covered by N-H bonds. Its intensity diminished following anti-thrombotic functionalization, which could be attributed to the presence of larger molecules on the outer layers. It is worth noting that atomic ratios should be compared between sub-peak components. Accordingly, the presence of nitrogenated species significantly decreased for both PC + HEP and PC + FPX. The findings substantiate the successful anti-thrombotic functionalization achieved via an indirect, *in-situ* reaction using GNPs as an intermediary.

#### 3.3.2 PEUU-COOH (PC)/anti-thrombotic functionalization FTIR spectroscopy analysis

Physicochemical evaluation by Fourier Transform analysis confirmed the presence of functional groups related to the functionalization of PC with polyethyleneimine (PC + PEI), PEG 4 ARM (PC + P4A), Seleno-L-Cystine (PC + SLC), Fondaparinux (PC + FPX) and Heparin (PC + HEP).

**FIGURE 8:**
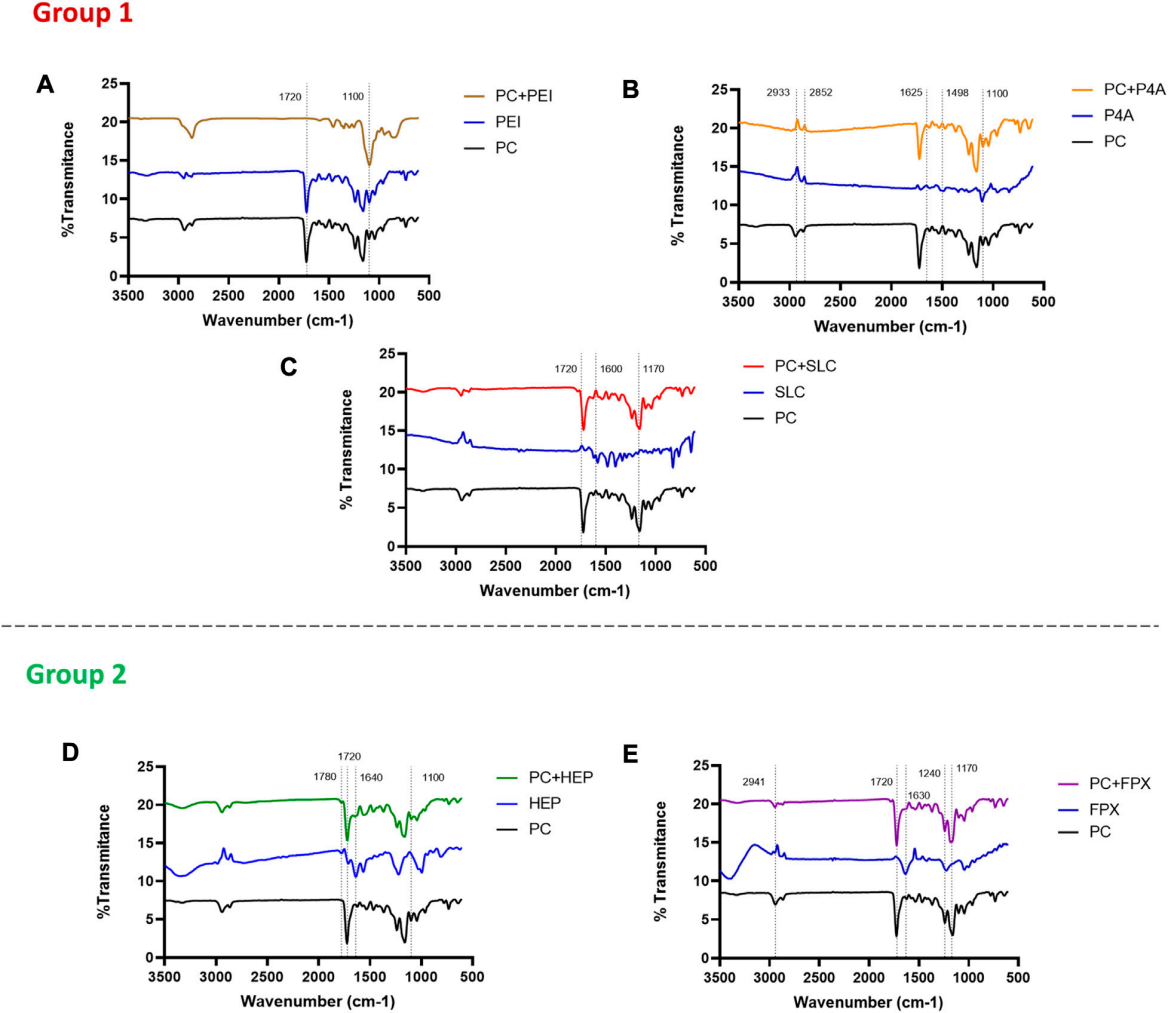
FTIR evaluation of the functionalization of PC. **(A)** FTIR spectra of PC functionalized with Polyethylenimine (PC + PEI), **(B)** FTIR spectra of PC functionalized with PEG 4 ARM Amine (PC + P4A), **(C)** FTIR spectra of PC functionalized with Seleno—L—Cystine (PC + SLC), **(D)** FTIR spectra of PC functionalized with HEP and **(E)** FTIR spectra of PC functionalized with FPX.

For group 1 molecules, the carbonyl group peak of the PC (1720 cm^-1^) exhibited attenuation, which is evident in PC + PEI, indicating functionalization, moreover, a decrease in absorption at 1720 cm^-1^ is also evident, a characteristic peak for imide carbonyl groups in PEI (Chen et al., 2006; Chen et al., 2006). Other identified peaks were located between 2950 and 2865 cm^-1^ (C-H stretching vibration) and decreased following PEI functionalization. Furthermore, the peak at 1100 cm^-1^ (stretching vibration of the ester group C-O-C) exhibited an increase. For PC + P4A, the C-H stretching vibration (2933 cm^-1^ to 2852 cm^-1^) and the amine group (1625 cm^-1^) stretching appeared more pronounced than in PC, suggesting that functionalization was successful. Conversely, the N-O group peak at 1498 cm^-1^ has decreased. Finally, the ether group at the 1100 cm^-1^ peak increased because of the formation of bonds during the functionalization process. Furthermore, a slight attenuation of the carbonyl group at 1720 cm^-1^ is observed at the time of functionalization, which indicates the bonding of the NH2 groups of P4A to PC. The characteristic peaks of PC + SLC include the carbonyl group peak of 1720 cm^-1^, where the PC + SLC polymer shows a decrease compared to PC. Similarly, the peak at 1600 cm^-1^, corresponding to the amine group, increased, and the peak of the ether group at 1170 cm^-1^ decreased (Figures 8A–C).

On the other hand, the analysis of group 2 molecules revealed notable peak changes, suggesting that Heparin (PC + HEP), and Fondaparinux (PC + FPX) functionalization led to a decrease (Figures 8D, E). For HEP functionalization, the characteristic peaks show a decrease in the peaks in 1720 cm^-1^ (corresponding to the vibration of the carbonyl stretching, C=O), 1640 cm^-1^ (corresponding to the vibration of amine stretching, N-H), and 1100 cm^-1^ (corresponding to the vibration of ester stretching, C-O-C) in comparison to the PC. This observation is consistent with the HEP control displaying higher inverse peaks. Additionally, a new peak emerged at 1780 cm^-1^ (corresponding to the vibration of carbonyl C=O) that was not present in the PC control but appeared in the PC + HEP functionalization. Similarly, FPX functionalization demonstrated peak reductions at 2941 cm^-1^ (corresponding to the C-H stretching), 1592 cm^-1^ (corresponding to nitro-compounds), 1240 cm^-1^, and 1154 cm^-1^ (corresponding to the ester group) when compared to the PC control. In contrast, the carbonyl stretching (C=O) peak (1720 cm^-1^) increased. The FTIR analysis substantial peak changes after each functionalization, which collectively suggest successful functionalization of the polymer surface with the anti-thrombotic molecules.

#### 3.3.3 PEUU-COOH (PC)/anti-thrombotic functionalization thermogravimetric analysis

**FIGURE 9:**
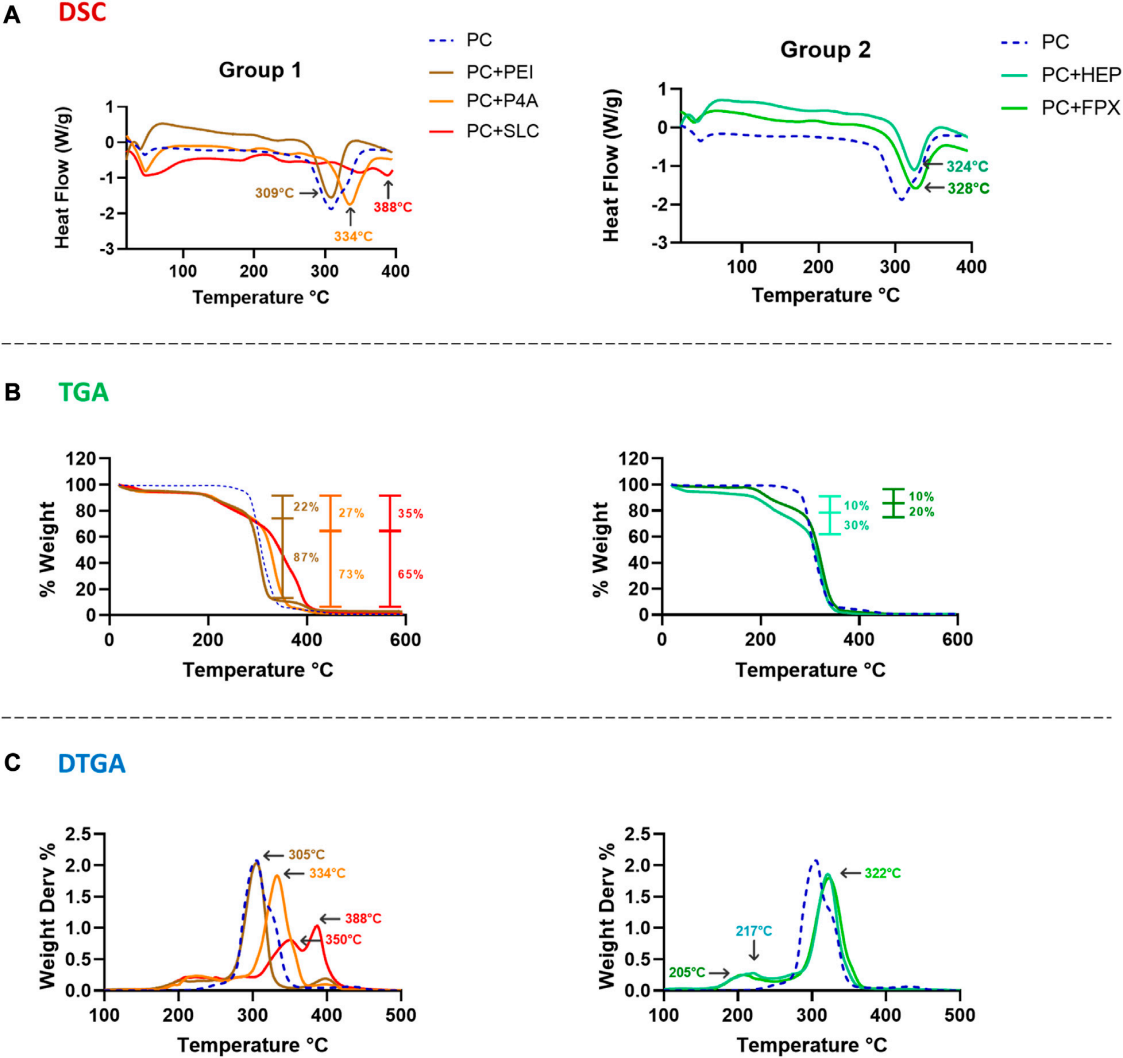
**(A)** DSC. **(B)** TGA. **(C)** DTGA results for PC *in-situ* functionalization (Blue dotted line) according to two different groups of bioactive molecules. Right column of data from Group 1–Direct Functionalization of PC + PEI (Brown), PC + P4A (Orange) and PC + SLC (Red). Left column of data from Group 2–Indirect Functionalization of PC + HEP (Aqua) and PC + FPX (Green).

DCS analysis of the different functionalization was performed to confirm the presence of active components. The DSC curves revealed that the melting temperatures (Tm) of functionalized polymers deviated from the PC control. In group 1, Tm for PC + PEI peaked at 309°C, for PC + P4A at 334°C, and for PC + SLC at 388°C. Similarly, in group 2, for PC + HEP the Tm peaked at 324°C while for PC + FPX at 328°C (Figure 9A).

TGA of functionalized polymers exhibited an initial weight loss at low temperatures due to the evaporation of water present in the sample. For group 1, varying percentages of weight loss were observed (Figure 9B). For PC + PEI and PC + P4A, two distinct percentages of weight loss were noted at 269°C and 322°C, which approached 22% and 87% for PC + PEI, since it has been found that pure PEI decomposition can occur at temperatures between 250°C and 350 °C (Zhu et al., 2007; Zhu et al., 2007), and weight loss at 305°C and 361°C which approached 30% and 93% for PC + P4A, in which the first weight loss is related to the decomposition temperature of P4A molecule, reported to be between 200°C and 300°C, and, the remaining percentage is associated with PC decomposition (Zarour et al., 2021; Zarour et al., 2021). A single weight loss of 35% at 317°C was observed for PC + SLC, which could be attributed to the rapid and spontaneous release of functionalizing molecules. This behavior might be explained by the detachment of each active molecule as the temperature increases, most likely reflecting the strength and the number of functionalized molecules on the surface.

In contrast, the degradation percentages for group 2 were higher than those of group 1 (Figure 9B), which might indicate the weight loss produced by the gelatin nanoparticles serving as bridges between the active molecule and the polymer. For PC + HEP, weight losses of 10% (180°C) and 36% (290°C) were observed, resulting from the degradation of the functionalization molecule and the gelatin nanoparticle. In the case of PC + FPX, a single weight loss of 23% (285°C) was observed, most likely due to the rapid degradation of both the gelatin nanoparticle and the FPX molecule. These results can be compared with the temperatures shown by the DTGA, where the points of inflection indicate the regimes of highest degradation of mass loss (Figure 9C). For PC + PEI and PC + P4A, the inflection points were 305°C and 334°C, respectively. On the other hand, the PC + SLC shows two important inflection points, 350°C and 388°C, which can be correlated to degradation events, leading to the release of material. This behavior is more evident in group 2, where bonding with gelatin likely causes a double inflection peak at 205°C and 322°C for PC + FPX. This is also the case of PC + HEP where the peaks appeared at 217°C and 322°C. This behavior can be similar to other articles that show similar temperature degradation against molecules such as HEP, PEG, and PEI (Kim et al., 2017; Kim et al., 2017; Zhu et al., 2021; Zhu et al., 2021).

### 3.4 PEUU-COOH (PC)/anti-thrombotic functionalization biocompatibility

**FIGURE 10:**
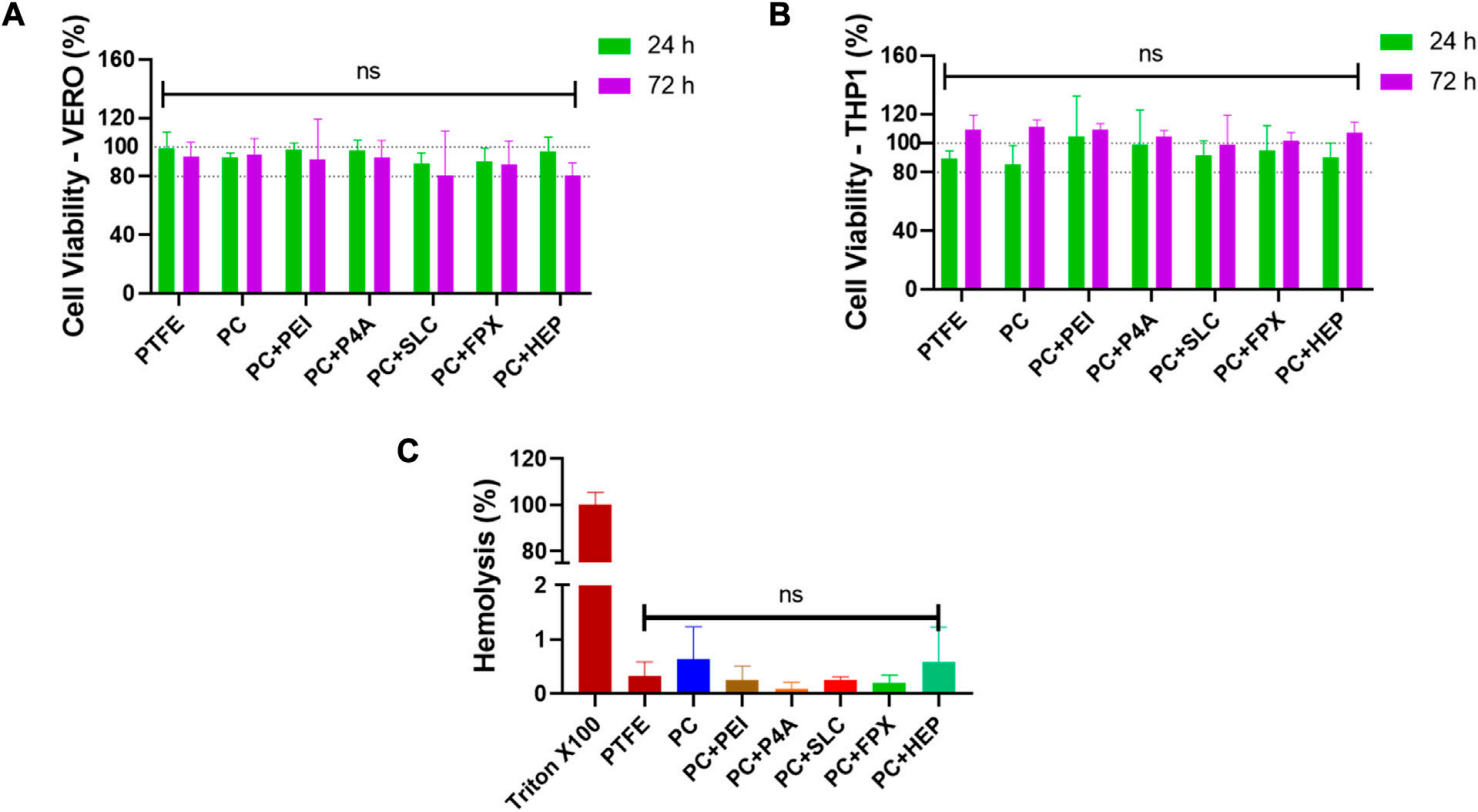
Viability percentage extracted from MTT data of tests performed by directly exposing **(A)** VERO cell cultures (ATCC CCL81.4) and **(B)** THP-1 Cell cultures (ATCC TIB-202) to film’s fragments for 24 h and 72 h. **(C)** Hemolytic behavior after an incubation time of 1 h. Triton X-100 and PBS were used as positive and negative controls respectively. Data compared to PC (PC) (Mean ± SD) where, ns = no significant *p ≤ 0.05 **p ≤ 0.01 ***p ≤ 0.001 ****p ≤ 0.0001.

The PC functionalization success with the anti-thrombotic molecules is contingent upon the biocompatibility of these biomaterials. The viability of VERO cells exposed directly to PC functionalized with the bioactive molecules was assessed at 24 and 72 h (Figure 10A). Results indicated that cell viability was maintained for all functionalized films. The highest viability percentages were observed for PC + PEI with 98% ± 28 at 24 h and 91% ± 28 at 72 h, followed by PC + P4A with 98% ± 12 at 24 h and 93% ± 12 at 72 h, and PC + HEP with 97% ± 9 at 24 h and 80% ± 9 at 72 h. Lower viability percentages were found for PC + FPX with 90% ± 16 at 24 h and 88% ± 15 at 72 h and PC + SLC with 89% ± 30 at 24 h and 80% ± 30 at 72 h. However, all groups exhibited a viability percentage above the minimum threshold specified by the 10993 ISO standard (80%), and no discernible cell morphological changes were observable. Similar behavior was found for THP-1 cells directly exposed to the films (Figure 10B). Higher viability rates were found for PC + PEI with 105% ± 27% and 109% ± 4 after 24 h and 72 h, followed by PC + P4A with 99% ± 23 and 104 ± 5 after 24 h and 72 h. Neither did the evaluated groups decrease the cell viability below 85%, nor did they show any statistically significant difference. Concurrently, the hemolysis percentage (Figure 10C) for all functionalized films remained below 1% without any statistically significant differences, considerably lower than the maximum percentage permitted by the 10993 ISO standard (5%).

### 3.5 PEUU-COOH (PC)/anti-thrombotic functionalization and anti-thrombogenic activity

**FIGURE 11:**
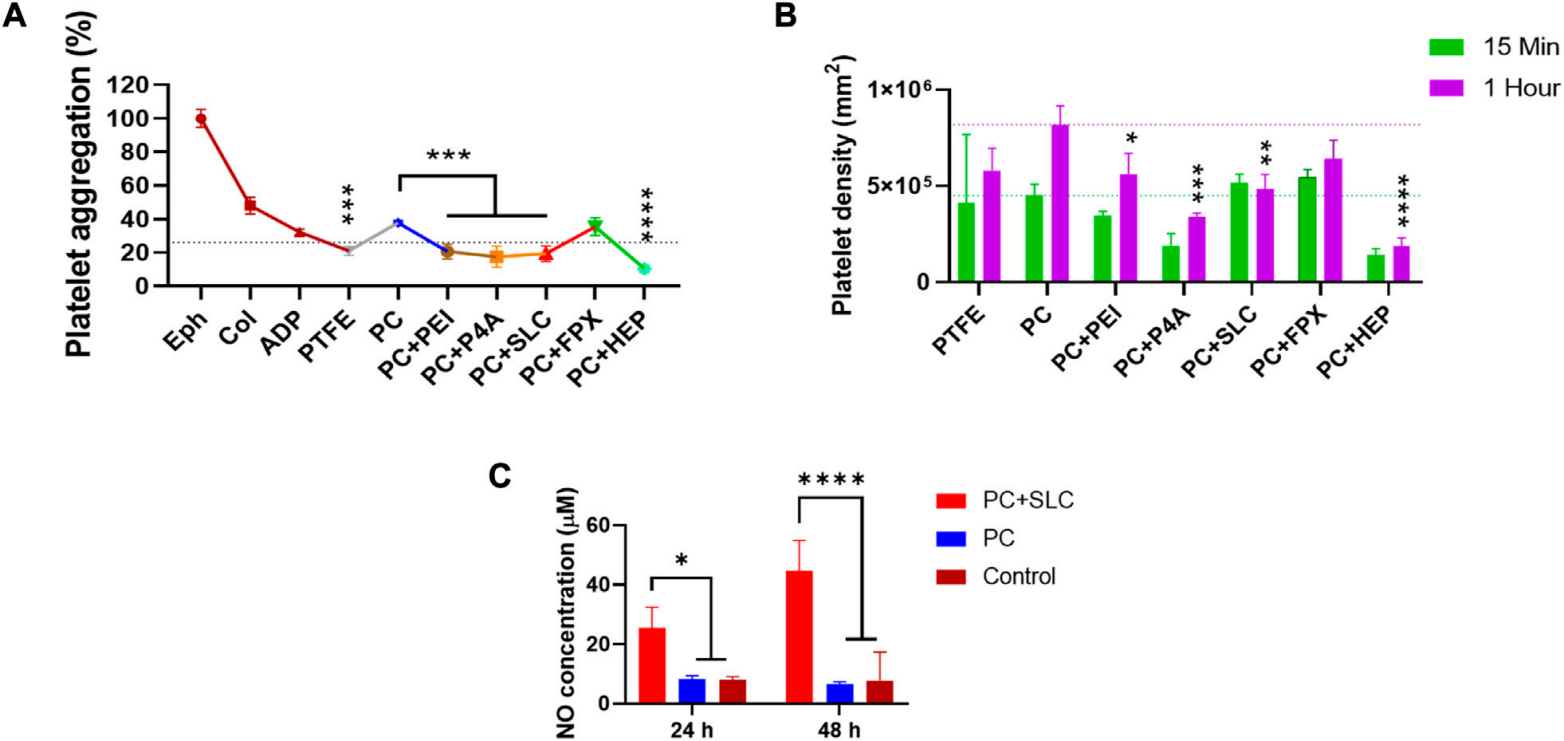
Platelet activation evaluation. **(A)** Platelet aggregation percentage, Epinephrine, Collagen, and ADP solutions were used as positive controls of high, medium, and low platelet aggregants. **(B)** Platelet density calculated as means of LDH release content of films exposed to platelets and treated with Triton X-100. **(C)** Concentration of nitric oxide species at 24 h and 48 h. Data compared to PC (Mean ± SD) where, ns = no significant *p ≤ 0.05 **p ≤ 0.01 ***p ≤ 0.001 ****p ≤ 0.0001.

An assessment of platelet aggregation percentages was conducted in light of the anticoagulant functionalization (Figure 11A). Films functionalized with epinephrine, collagen, and ADP were used as high, medium, and mild platelet aggregation inducers, respectively. In general, the anti-thrombotic functionalization led to a decreased platelet aggregation percentage compared to PEUU-COOH alone (37% ± 1). The lowest aggregation percentage was observed for PC + HEP, which registered 10% ± 2 (*p* ≤ 0.0001), followed by PC + P4A at 17% ± 6 (*p* ≤ 0.001), PC + SLC at 19% ± 5 (*p* ≤ 0.001) and PC + PEI at 20% ± 4 (*p* ≤ 0.001). Unexpectedly, PC + FPX presented a higher platelet aggregation percentage of 35% ± 5%, which was higher than that of PEUU-COOH.

To correlate these findings with the platelet adhesion and activation on the polymer surfaces, an LDH activation test was performed, and the total count of adhered platelets was calculated through a linear regression from a calibration curve (Figure 11B). The results indicated that the number of platelets adhering to the film surface was directly proportional to time. Platelet density increased after 1 h of exposure to PRP for all groups, except for SLC. According to the platelet aggregation test at 1 h, PC + HEP presented the lowest platelet count of 18022 2 ± 41460/mm^2^ (*p* ≤ 0.0001) followed by PC + P4A with 3 × 10^5^ ± 2 × 10^4^/mm^2^ (*p* ≤ 0.001), PC + SLC with 4 × 10^5^ ± 7 × 10^4^/mm^2^ (*p* ≤ 0.01) and PC + PEI with 5 × 10^5^ ± 1 × 10^5^/mm^2^ (*p* ≤ 0.05). Higher platelet counts were found for PC + FPX with 6 × 10^5^ ± 9 × 10^4^/mm^2^, with no significant difference from PC.

**FIGURE 12:**
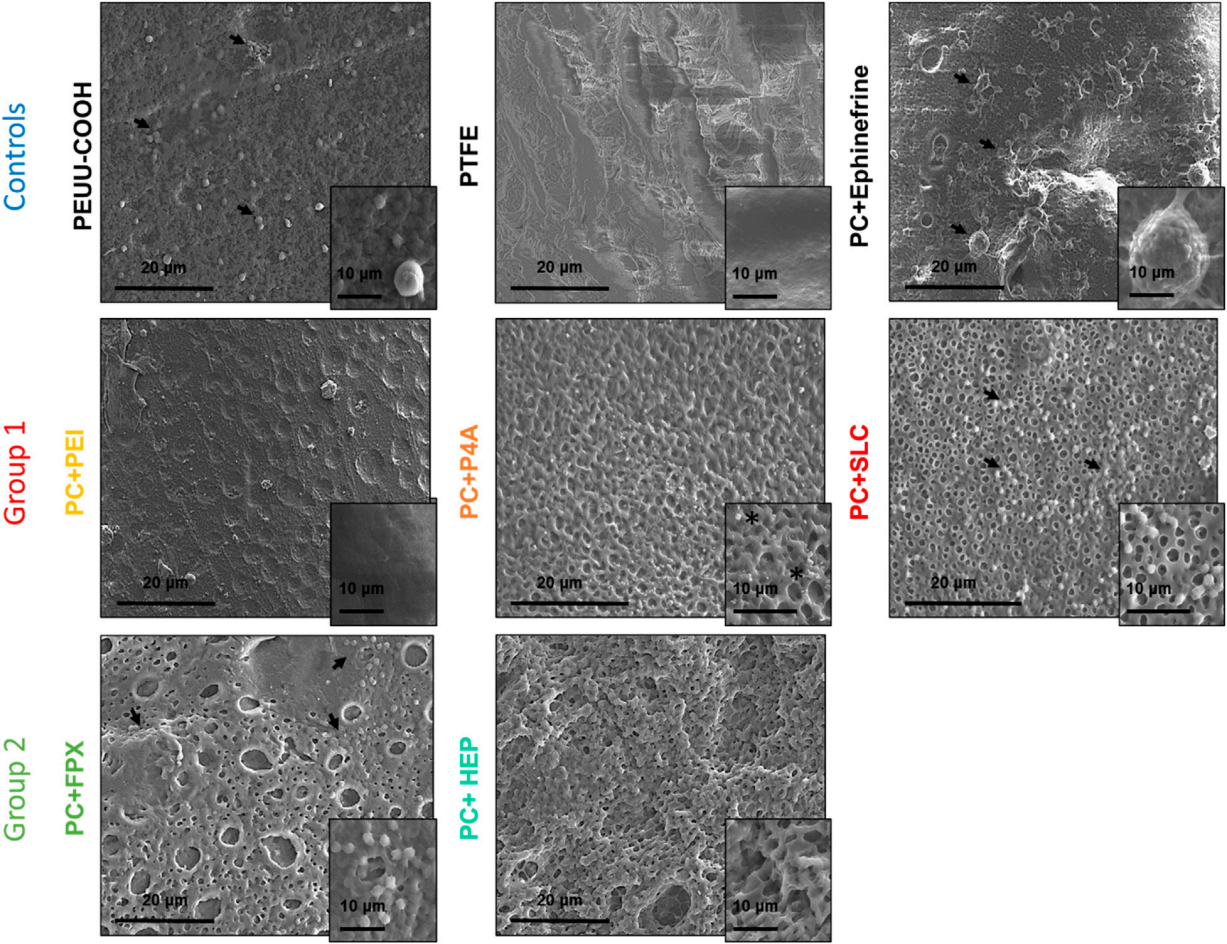
SEM microscopy images of the film’s surfaces exposed to PRP, the changes in topography and platelet adhesion and activation can be observed. Arrows show platelets, asterisks indicate salt crystals.

Platelet activation might be observed by SEM images (Figure 12). The surface functionalized with epinephrine showed a high platelet density, and platelets displayed filopodia, indicating attachment and activation. Similarly, PC showed high platelet density, albeit without filopodia. In contrast, a fragment of a PTFE vascular graft was presented without platelets. Regarding the anti-thrombotic activity functionalization, the surfaces of the functionalized polymers appeared markedly different from the PC alone. A highly porous surface was observed for PC + FPX, less porous surfaces for PC + HEP and PC + P4A, and smoother surfaces for PC + SLC and PC + PEI. Platelets were found to attach on PC + FPX and PC + SLC, whereas PC + HEP, PC + P4A, and PC + PEI showed no adhered platelets. On the other hand, given that the SLC action process is attributed to the production of reactive nitric oxide species, its efficacy was verified by quantifying the NO2-/NO3- species ratio. Nitric oxide at 24 and 48 h was evaluated for PC + SLC to confirm its activity (Figure 11C), and results indicated an increase from 25 uM to 45 uM, almost doubling the amount at 24 h. This is most likely a result of the enhanced production of nitric oxide species due to PC-SLC activity as an organoselenium compound, this might lead to elevated cyclic guanosine monophosphate levels and decreasing Ca^2+^ levels, which are required for platelet activation in the coagulation process (Suchyta et al., 2014; Suchyta et al., 2014).

As shown above, the most potent anticoagulant activity was achieved for heparin-functionalized materials followed by PEG-4-Arm-NH2 and PEI. Surprisingly, Seleno-L-cystine and Fondaparinux showed no anti-platelet activity. These results align with the literature, in which the two molecules showing the most significant anti-platelet effects are Heparin and various PEG polymers such as the PEG-4-Arm (Ashcraft et al., 2021; Ashcraft et al., 2021). These compounds shown to be effective because they neutralize the prostaglandin prostacyclin, a potent inhibitor of platelet aggregation that can also inhibit integrin exposure. Additionally, the PEG-4-arm used here is of a longer chain. As previously mentioned, increased PEG length reduces protein adsorption and cell adhesion capabilities, which is beneficial for controlling cell adhesion and inflammation (J.-L. Wang et al., 2018). This technique has also been previously reported as a passivation strategy to inhibit unspecific surface interaction (Sauter et al., 2013).

**FIGURE 13:**
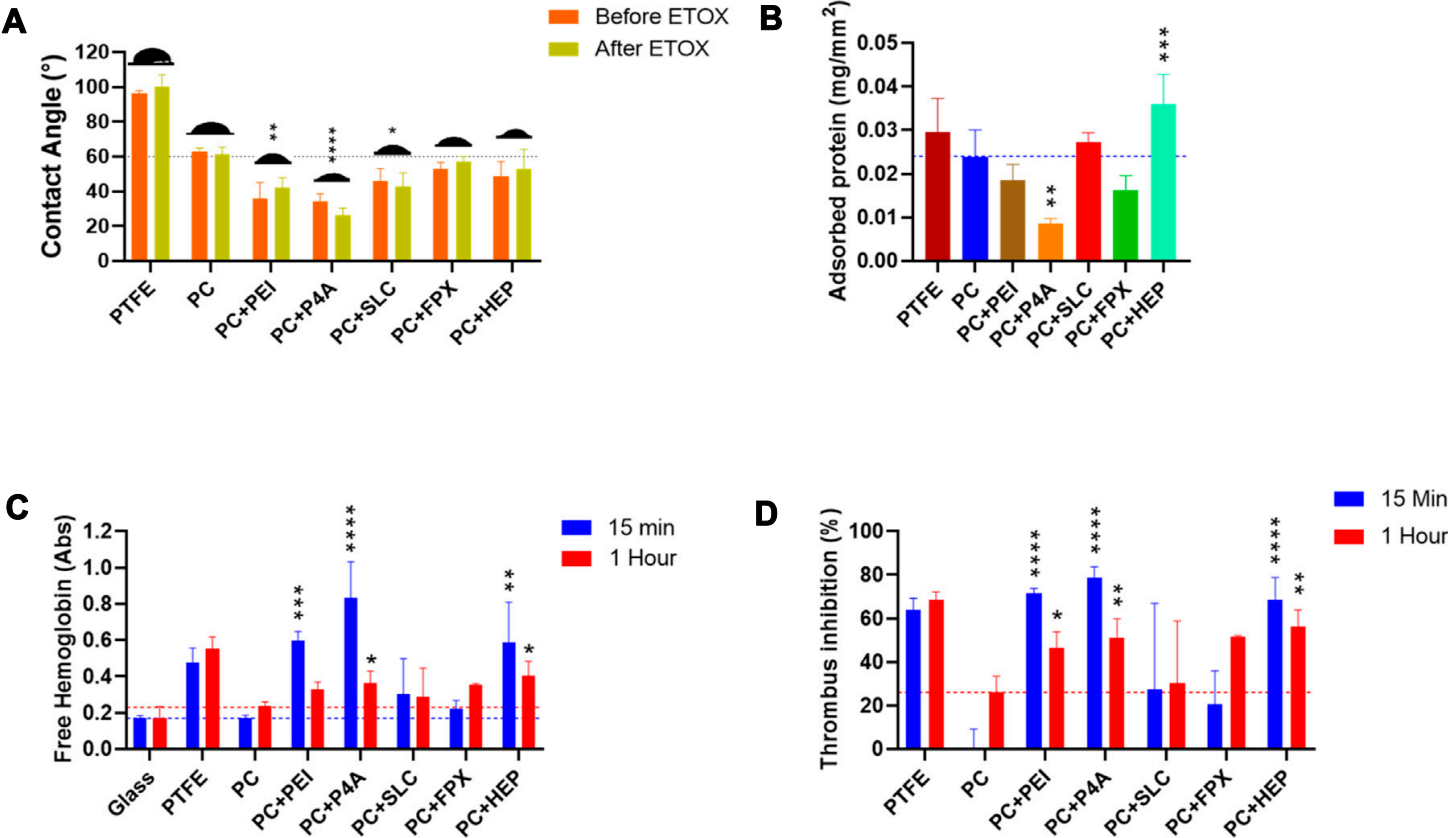
Antithrombogenic activity evaluation. **(A)** Contact angle of the surface of the functionalized films. **(B)** Adsorbed protein over the surface of the functionalized films. **(C)** Free hemoglobin released of clots formed on the surfaces of the films and exposed to water at 15 and 1 h, glass and PTFE were used as negative and positive controls. **(D)** Thrombus Inhibition percentage extracted from free hemoglobin and considering the PC value as the initial condition.

For a more detailed analysis of the anti-thrombotic properties of the surface-functionalized films, we measured the contact angle and protein adsorption. Furthermore, free hemoglobin and thrombus inhibition tests were performed. As anticipated, Surface functionalization with P4A and PEI, given their inherent hydrophilicity, reduced the contact angle on PEUU-COOH (Figure 13A) with 34° ± 6 for PC + P4A (*p* ≤ 0.001) and 36 ± 9 for PC + PEI (*p* ≤ 0.001). However, post-ethylene oxide sterilization, a slight decrease for PC + P4A and an increase for PC + PEI were noted, with both changes lacking statistical significance, i.e., 26° ± 4 and 42° ± 5, respectively. Functionalization with SLC and HEP also resulted in minor contact angle reductions with 46° ± 7 and 42° ± 8 (*p* ≤ 0.05) and 49° ± 8 and 52 ± 8 (*p* ≤ 0.05) pre- and post-sterilization for PC + SLC and PC + HEP, respectively. In contrast, PC + FPX contact angle remained relatively stable (53° ± 4 and 57° ± 3 pre- and post-sterilization). Accordingly, adsorbed protein for PC was determined to be 2 × 10^−2^ ± 6 × 10^−3^ mg/mm^2^ (Figure 13B) and the protein adsorbed on the PC + P4A film was 8 × 10^−3^ ± 1 × 10^−3^ mg/mm^2^ (*p* ≤ 0.01). Contrary to expectations, PC + HEP exhibited even higher adsorbed protein levels than PC, with 3 × 10^−2^ ± 6 × 10^−3^ mg/mm^2^ (*p* ≤ 0.001). No other statistically significant differences were found among the groups. Furthermore, it is noteworthy that both PC + PEI and PC + P4A formulations exhibited a marginal decline in protein adsorption, aligning with reduced hemolysis percentages. Conventionally, the positive charge of PEI and P4A surfaces, due to their terminal amine groups, would suggest enhanced protein adsorption. However, the observed attenuation in protein adsorption could conceivably hint at potential amide bond formation between the carboxyl groups of PC and the amine groups of PEI and P4A, possibly leading to a muted positive charge on these surfaces. Concurrently, this diminished positive charge is postulated to boost hemocompatibility, a phenomenon attributable to the reduced interaction between the polymer and negatively charged erythrocytes (Monfared et al., 2022; S; Zhu et al., 2007).

The free hemoglobin tests were performed to measure the amount of free hemoglobin released due to reduced coagulation processes. Higher absorbance values correlate with lower coagulation potential and increased hemocompatibility. After exposing the films to whole and activated blood for 15 min, free hemoglobin levels were recorded. In agreement with previous experiments, PC + P4A showed the highest absorbance for free hemoglobin with 0.8 ± 0.2 (*p* ≤ 0.0001), compared to PC alone with 0.2 ± 3 × 10^−3^, followed by PC + PEI with 0.6 ± 5 × 10^−2^ (*p* ≤ 0.001) and PC + HEP with 0.58 ± 0.2 (*p* ≤ 0.01). The behavior persisted for 1 h, during which the PC + P4A and PC + HEP maintained their anticoagulant effects. No other statistically significant differences were found among the groups (Figure 13C).

To further analyze data, thrombus generation was induced on a glass surface, which was considered the maximum value for coagulation. Accordingly, thrombus inhibition percentages were derived from the free hemoglobin test. Consistent data showed the highest thrombus inhibition percentage achieved by PC + P4A with 79% ± 5 (*p* ≤ 0.0001) followed by PC + PEI with 71% ± 2 (*p* ≤ 0.0001) and PC + HEP with 69% ± 10 (*p* ≤ 0.0001). This behavior persisted for up to 1 h for these groups. Nevertheless, PC + FPX did not exhibit a significant increase in the thrombus inhibition percentage, and PC + SLC displayed a highly variable behavior (Figure 13D).

## 4 Conclusion

The development of innovative surface modifications to enhance the anti-thrombotic performance of Polyester Urethane Urea (PEUU) is critical for advancing regenerative cardiovascular devices. In this study, a biodegradable PEUU elastomer with carboxyl groups was synthesized to facilitate functionalization with anti-thrombotic molecules. The carboxylation of PEUU proved successful, maintaining excellent biocompatibility with cellular viability above 85% and hemolysis percentage below 2%, in accordance with international ISO10993 standards. Various strategies were employed to functionalize the carboxylated PEUU, targeting different biochemical routes to inhibit platelet adhesion, activation, or activity. These included the use of PEI (400 MW) and PEG-4-arm (20000 MW) to induce steric inhibition, Seleno-L-Cystine as a Nitric Oxide producer, Fondaporinux as a selective factor Xa inhibitor, and Heparin as an inhibitor of both factor Xa and thrombin. Physicochemical characterization confirmed successful functionalization and excellent biocompatibility for all bioactive molecules.

The most effective anti-thrombotic results were achieved with PC functionalized with Heparin and PEG-4-arm, significantly reducing platelet adhesion, and demonstrating high thrombus inhibition. While PEI and Seleno-L-Cystine functionalization exhibited anti-thrombotic effects, they were not as potent. Surprisingly, Fondaporinux functionalization did not yield any significant improvement in thrombogenesis. Future applications of synthesized PEUU functionalized with anti-thrombotic molecules could include anti-thrombogenic biomaterials for cardiovascular devices such as tissue-engineered vascular grafts, synthetic vascular graft coatings, stents, valves, or catheters. Additionally, PC functionalization with other bioactive molecules could broaden its potential biomedical applications.

All in all, the development of anti-thrombotic-functionalized PEUU offers promising advancements for regenerative cardiovascular devices by improving hemocompatibility and reducing thrombogenesis. However, it is essential to consider the tradeoffs between various functionalization strategies and the challenges associated with different approaches to optimize outcomes.

## 5 Patents

Biodegradable, Non-Thrombogenic Elastomeric Polyurethanes. Patent Application Publication, United States. Pub. No: US 2014/0248232 A^1^. Pub. Date: Sep. 4, 20164.

## Data Availability

The original contributions presented in the study are included in the article/Supplementary Material, further inquiries can be directed to the corresponding author.

## References

[B1] An J. Chen S. Gao J. Zhang X. Wang Y. Li Y. (2015). Construction and evaluation of nitric oxide generating vascular graft material loaded with organoselenium catalyst via layer-by-layer self-assembly. Sci. China Life Sci. 58 (8), 765–772. 10.1007/s11427-015-4870-z 26014212

[B2] Ashcraft M. Douglass M. Chen Y. Handa H. (2021). Combination strategies for antithrombotic biomaterials: an emerging trend towards hemocompatibility. Biomaterials Sci. 9 (7), 2413–2423. 10.1039/D0BM02154G PMC8035307 33599226

[B3] Babaev M. S. Lobov A. N. Shishlov N. M. Kolesov S. V. (2022). pH-sensitive particles of polymer-surfactant complexes based on a copolymer of N,N′-diallyl-N,N′-dimethylammonium chloride with maleic acid and sodium dodecyl sulfate. React. Funct. Polym. 178, 105359. 10.1016/j.reactfunctpolym.2022.105359

[B4] Barbosa L. C. A. Maltha C. R. A. Demuner A. J. Cazal C. M. Reis E. L. Colodette J. L. (2013). A rapid method for quantification of carboxyl groups in cellulose pulp. BioResources 8 (1). 10.15376/biores.8.1.1043-1054

[B5] Bednarz P. Bernasik A. Laska J. (2018). The influence of sterilization on properties of polyurethane/polylactide blend. Sci. Technol. Innovation 2 (1), 13–18. 10.5604/01.3001.0012.1264

[B6] Braune S. Zhou S. Groth B. Jung F. (2015). Quantification of adherent platelets on polymer-based biomaterials. Comparison of colorimetric and microscopic assessment. Clin. Hemorheol. Microcirc. 61 (2), 225–236. 10.3233/CH-151995 26410874

[B7] Chen B.-K. Su C.-T. Tseng M.-C. Tsay S.-Y. (2006). Preparation of polyetherimide nanocomposites with improved thermal, mechanical and dielectric properties. Polym. Bull. 57 (5), 671–681. 10.1007/s00289-006-0630-3

[B8] Chu A. J. Beydoun S. Mathews S. T. Hoang J. (2003). Novel anticoagulant polyethylenimine: inhibition of thrombin-catalyzed fibrin formation. Archives Biochem. Biophysics 415 (1), 101–108. 10.1016/S0003-9861(03)00216-9 12801518

[B9] Ed Rainger G. Chimen M. Harrison M. J. Yates C. M. Harrison P. Watson S. P. (2015). The role of platelets in the recruitment of leukocytes during vascular disease. Platelets 26 (6), 507–520. 10.3109/09537104.2015.1064881 26196409 PMC4673595

[B10] El Jundi A. Buwalda S. Bethry A. Hunger S. Coudane J. Bakkour Y. (2020). Double-hydrophilic block copolymers based on functional poly(ε-caprolactone)s for pH-dependent controlled drug delivery. Biomacromolecules 21 (2), 397–407. 10.1021/acs.biomac.9b01006 31571489

[B11] El-Raheem H. A. Hassan R. Y. A. Khaled R. Farghali A. El-Sherbiny I. M. (2021). New sensing platform of poly(ester-urethane)urea doped with gold nanoparticles for rapid detection of mercury ions in fish tissue. RSC Adv. 11 (50), 31845–31854. 10.1039/D1RA03693A 35496891 PMC9041571

[B12] Fang J. Ye S.-H. Shankarraman V. Huang Y. Mo X. Wagner W. R. (2014). Biodegradable poly(ester urethane)urea elastomers with variable amino content for subsequent functionalization with phosphorylcholine. Acta Biomater. 10 (11), 4639–4649. 10.1016/j.actbio.2014.08.008 25132273 PMC4655827

[B13] Gonzalez-Melo C. Garcia-Brand A. J. Quezada V. Reyes L. H. Muñoz-Camargo C. Cruz J. C. (2021). Highly efficient synthesis of type B gelatin and low molecular weight chitosan nanoparticles: potential applications as bioactive molecule carriers and cell-penetrating agents. Polymers 13 (23), 4078. 10.3390/polym13234078 34883582 PMC8659274

[B14] Guan J. Sacks M. S. Beckman E. J. Wagner W. R. (2002). Synthesis, characterization, and cytocompatibility of elastomeric, biodegradable poly(ester-urethane)ureas based on poly(caprolactone) and putrescine. J. Biomed. Mater. Res. 61 (3), 493–503. 10.1002/jbm.10204 12115475

[B15] Guidoin R. Canizales S. Charara J. How T. Marois Y. Batt M. (1992). Vascular access for hemodialysis: pathologic features of surgically excised ePTFE grafts. Ann. Vasc. Surg. 6 (6), 517–524. 10.1007/BF02000823 1463665

[B16] Halbert R. J. Nicholson G. Nordyke R. J. Pilgrim A. Niklason L. (2020). Patency of ePTFE arteriovenous graft placements in hemodialysis patients: systematic literature review and meta-analysis. Kidney360 1 (12), 1437–1446. 10.34067/KID.0003502020 35372887 PMC8815525

[B17] Hong Y. Ye S.-H. Pelinescu A. L. Wagner W. R. (2012). Synthesis, characterization, and paclitaxel release from a biodegradable, elastomeric, poly(ester urethane)urea bearing phosphorylcholine groups for reduced thrombogenicity. Biomacromolecules 13 (11), 3686–3694. 10.1021/bm301158j 23035885 PMC3839290

[B18] Hu C. Luo R. Wang Y. (2020). Heart valves cross-linked with erythrocyte membrane drug-loaded nanoparticles as a biomimetic strategy for anti-coagulation, anti-inflammation, anti-calcification, and endothelialization. ACS Appl. Mater. Interfaces 12 (37), 41113–41126. 10.1021/acsami.0c12688 32833422

[B19] International Organization for Standardization (2017). ISO 10993-4:2017 - biological evaluation of medical devices — Part 4: Selection of tests for interactions with blood. Geneva, Switzerland: The ISO Centra Secretariat.

[B20] Irvine S. A. Yun X. Venkatraman S. (2012). Anti-platelet and tissue engineering approaches to biomaterial blood compatibilization: how well have these been translated into the clinic? Drug Deliv. Transl. Res. 2 (5), 384–397. 10.1007/s13346-012-0077-z 25787176

[B21] Janairo R. R. R. Henry J. J. D. Lee B. L.-P. Hashi C. K. Derugin N. Lee R. (2012). Heparin-modified small-diameter nanofibrous vascular grafts. IEEE Trans. NanoBioscience 11 (1), 22–27. 10.1109/TNB.2012.2188926 22434651

[B22] Kim M. Lin M. M. Sohn Y. Kim J. Kang B. S. Kim D. K. (2017). Polyethyleneimine-associated polycaprolactone—superparamagnetic iron oxide nanoparticles as a gene delivery vector. J. Biomed. Mater. Res. Part B Appl. Biomaterials 105 (1), 145–154. 10.1002/jbm.b.33519 26443109

[B23] Liang N. L. Baril D. T. Avgerinos E. D. Leers S. A. Makaroun M. S. Chaer R. A. (2017). Comparative effectiveness of anticoagulation on midterm infrainguinal bypass graft patency. J. Vasc. Surg. 66 (2), 499–505.e2. 10.1016/j.jvs.2016.12.141 28400219 PMC5524600

[B24] Liang X. Hu C. Wang Y. (2023). Biomimetic-modified bioprosthetic heart valves with controlled release of glycyrrhizin acid mediated by the inflammatory microenvironment for anti-thrombotic, anti-inflammatory, and anti-calcification. Chem. Eng. J. 472, 145044. 10.1016/j.cej.2023.145044

[B25] Liu R. Qin Y. Wang H. Zhao Y. Hu Z. Wang S. (2013). The *in vivo* blood compatibility of bio-inspired small diameter vascular graft: effect of submicron longitudinally aligned topography. BMC Cardiovasc. Disord. 13 (1), 79. 10.1186/1471-2261-13-79 24083888 PMC3850682

[B26] Monfared Y. K. Mahmoudian M. Cecone C. Caldera F. Haiaty S. Heidari H. R. (2022). Hyper-branched cationic cyclodextrin polymers for improving plasmid transfection in 2D and 3D spheroid cells. Pharmaceutics 14 (12), 2690. 10.3390/pharmaceutics14122690 36559184 PMC9785855

[B27] Nair P. A. Ramesh P. (2013). Electrospun biodegradable calcium containing poly(ester-urethane)urea: synthesis, fabrication, *in vitro* degradation, and biocompatibility evaluation. J. Biomed. Mater. Res. Part A 101A (7), 1876–1887. 10.1002/jbm.a.34490 23712992

[B28] Poussard L. Burel F. Couvercelle J.-P. Merhi Y. Tabrizian M. Bunel C. (2004). Hemocompatibilty of new ionic polyurethanes: influence of carboxylic group insertion modes. Biomaterials 25 (17), 3473–3483. 10.1016/j.biomaterials.2003.10.069 15020121

[B29] Pozzi D. Colapicchioni V. Caracciolo G. Piovesana S. Capriotti A. L. Palchetti S. (2014). Effect of polyethyleneglycol (PEG) chain length on the bio–nano-interactions between PEGylated lipid nanoparticles and biological fluids: from nanostructure to uptake in cancer cells. Nanoscale 6 (5), 2782. 10.1039/c3nr05559k 24463404

[B30] Ramachandran B. Chakraborty S. Dixit M. Muthuvijayan V. (2018). A comparative study of polyethylene terephthalate surface carboxylation techniques: characterization, *in vitro* haemocompatibility and endothelialization. React. Funct. Polym. 122, 22–32. 10.1016/j.reactfunctpolym.2017.11.001

[B31] Rodriguez-Soto M. A. Suarez Vargas N. Riveros A. Camargo C. M. Cruz J. C. Sandoval N. (2021). Failure analysis of TEVG’s I: overcoming the initial stages of blood material interaction and stabilization of the immune response. Cells 10 (11), 3140. 10.3390/cells10113140 34831361 PMC8625197

[B32] Sabino R. Popat K. (2020). Evaluating whole blood clotting *in vitro* on biomaterial surfaces. BIO-PROTOCOL 10 (3), e3505. 10.21769/BioProtoc.3505 33654732 PMC7842529

[B33] Sauter A. Richter G. Micoulet A. Martinez A. Spatz J. P. Appel S. (2013). Effective polyethylene glycol passivation for the inhibition of surface interactions of peripheral blood mononuclear cells and platelets. Biointerphases 8 (1), 14. 10.1186/1559-4106-8-14 24706127 PMC5849213

[B34] Schoenrath F. Just I. A. Falk V. Emmert M. Y. (2021). Antiplatelet and direct oral anticoagulation management after coronary artery bypass graft surgery: the cinderella of current cardiovascular trials, please show me (some) evidence. Eur. Heart J. 42 (22), 2145–2148. 10.1093/eurheartj/ehab033 33615338

[B35] Shen X. Fang J. Lv X. Pei Z. Wang Y. Jiang S. (2011). Heparin impairs angiogenesis through inhibition of MicroRNA-10b. J. Biol. Chem. 286 (30), 26616–26627. 10.1074/jbc.M111.224212 21642433 PMC3143626

[B36] Shiu H. T. Goss B. Lutton C. Crawford R. Xiao Y. (2014). Controlling whole blood activation and resultant clot properties by carboxyl and alkyl functional groups on material surfaces: a possible therapeutic approach for enhancing bone healing. J. Mater. Chem. B 2 (20), 3009–3021. 10.1039/C4TB00009A 32261676

[B37] Stavrou E. Schmaier A. H. (2010). Factor XII: what does it contribute to our understanding of the physiology and pathophysiology of hemostasis and thrombosis. Thrombosis Res. 125 (3), 210–215. 10.1016/j.thromres.2009.11.028 PMC2851158 20022081

[B38] Suchyta D. J. Handa H. Meyerhoff M. E. (2014). A nitric oxide-releasing heparin conjugate for delivery of a combined antiplatelet/anticoagulant agent. Mol. Pharm. 11 (2), 645–650. 10.1021/mp400501c 24423090 PMC3993940

[B39] Tang C. Kligman F. Larsen C. C. Kottke-Marchant K. Marchant R. E. (2009). Platelet and endothelial adhesion on fluorosurfactant polymers designed for vascular graft modification. J. Biomed. Mater. Res. Part A 88A (2), 348–358. 10.1002/jbm.a.31888 PMC3147223 18286624

[B40] Tousoulis D. Kampoli A.-M. Tentolouris Nikolaos Papageorgiou C. Stefanadis C. (2012). The role of nitric oxide on endothelial function. Curr. Vasc. Pharmacol. 10 (1), 4–18. 10.2174/157016112798829760 22112350

[B41] Valencia-Rivero K. T. Cruz J. C. Wagner-Gutierrez N. D’Amore A. Miranda M. C. López R. (2019). Evaluation of microscopic Structure−Function relationships of PEGylated small intestinal submucosa vascular grafts for arteriovenous connection. ACS Appl. Bio Mater. 2 (9), 3706–3721. 10.1021/acsabm.9b00158 35021344

[B42] Wang G.-R. Zhu Y. Halushka P. V. Lincoln T. M. Mendelsohn M. E. (1998). Mechanism of platelet inhibition by nitric oxide: *in vivo* phosphorylation of thromboxane receptor by cyclic GMP-dependent protein kinase. Proc. Natl. Acad. Sci. 95 (9), 4888–4893. 10.1073/pnas.95.9.4888 9560198 PMC20183

[B43] Wang J.-L. Du X.-J. Yang J.-X. Shen S. Li H.-J. Luo Y.-L. (2018). The effect of surface poly(ethylene glycol) length on *in vivo* drug delivery behaviors of polymeric nanoparticles. Biomaterials 182, 104–113. 10.1016/j.biomaterials.2018.08.022 30114562

[B44] Włoch M. Datta J. Błażek K. (2018). The effect of high molecular weight bio-based diamine derivative of dimerized fatty acids obtained from vegetable oils on the structure, morphology and selected properties of poly(ether-urethane-urea)s. J. Polym. Environ. 26 (4), 1592–1604. 10.1007/s10924-017-1059-5

[B45] Yang J. Welby J. L. Meyerhoff M. E. (2008). Generic nitric oxide (NO) generating surface by immobilizing organoselenium species via layer-by-layer assembly. Langmuir 24 (18), 10265–10272. 10.1021/la801466e 18710268 PMC2824255

[B46] Yau J. W. Teoh H. Verma S. (2015). Endothelial cell control of thrombosis. BMC Cardiovasc. Disord. 15 (1), 130. 10.1186/s12872-015-0124-z 26481314 PMC4617895

[B47] Zarour A. Omar S. Abu-Reziq R. (2021). Preparation of poly(ethylene glycol)@Polyurea microcapsules using oil/oil emulsions and their application as microreactors. Polymers 13 (15), 2566. 10.3390/polym13152566 34372169 PMC8348332

[B48] Zhang Y. Zhang M. Tan L. Pan N. Zhang L. (2019). The clinical use of fondaparinux: A synthetic heparin pentasaccharide. Prog. Mol. Biol. Transl. Sci. 163, 41–53. 10.1016/bs.pmbts.2019.02.004 31030756

[B49] Zhou H. Xun R. Liu Q. Fan H. Liu Y. (2014). Preparation of thermal and pH dually sensitive polyurethane membranes and their properties. J. Macromol. Sci. Part B 53 (3), 398–411. 10.1080/00222348.2013.845059

[B50] Zhu S. Qian F. Zhang Y. Tang C. Yin C. (2007). Synthesis and characterization of PEG modified N-trimethylaminoethylmethacrylate chitosan nanoparticles. Eur. Polym. J. 43 (6), 2244–2253. 10.1016/j.eurpolymj.2007.03.042

[B51] Zhu T. Gu H. Zhang H. Wang H. Xia H. Mo X. (2021). Covalent grafting of PEG and heparin improves biological performance of electrospun vascular grafts for carotid artery replacement. Acta Biomater. 119, 211–224. 10.1016/j.actbio.2020.11.013 33181359

[B52] Zhu X. Uchikoshi T. Suzuki T. S. Sakka Y. (2007). Effect of polyethylenimine on hydrolysis and dispersion properties of aqueous Si 3 N4 suspensions. J. Am. Ceram. Soc. 90 (3), 797–804. 10.1111/j.1551-2916.2007.01491.x

