# Supplementary material for: Polyester urethane urea (PEUU) functionalization for enhanced anti-thrombotic performance: advancing regenerative cardiovascular devices through innovative surface modifications
Source: Front Bioeng Biotechnol. 2023 Sep 20;11:1257778. doi: 10.3389/fbioe.2023.1257778 (PMC10548217; doi:10.3389/fbioe.2023.1257778)
Supplement: Supplementary file 1 [file Table1.DOCX]

Supplementary Material

Polyester Urethane Urea (PEUU) Functionalization for Enhanced Antiplatelet Performance: Advancing Regenerative Cardiovascular Devices through Innovative Surface Modifications.

**María A. Rodríguez Soto^1^, Natalia Suarez Vargas^1^, María Ayala-Velásquez^1^, Andrés M. Aragón-Rivera^1^, Carlos Ostos^2^, Juan C. Cruz^1^, Carolina Muñoz Camargo^1^, Seungil Kim^3^, Antonio D’amore^3^, William R. Wagner^3^, Juan C. Briceño^1,4^.**

^1^Department of Biomedical Engineering, Universidad de los Andes, Cra. 1E No. 19a – 40, Bogotá, DC 111711, Colombia. [Ma.rodriguezs1@uniandes.edu.co](mailto:Ma.rodriguezs1@uniandes.edu.co) (M.A.R-S); [na.suarez122@uniandes.edu.co](mailto:na.suarez122@uniandes.edu.co) (N.S.V); [md.ayala@uniandes.edu.co](mailto:md.ayala@uniandes.edu.co) (M.A-V); [am.aragonr@uniandes.edu.co](mailto:am.aragonr@uniandes.edu.co) (A.M.A-R); [jc.cruz@uniandes.edu.co](mailto:jc.cruz@uniandes.edu.co) (J.C.C); [c.munoz2016@uniandes.edu.co](mailto:c.munoz2016@uniandes.edu.co) (C.M.C); [jbriceno@uniandes.edu.co](mailto:jbriceno@uniandes.edu.co) (J.C.B)

^2^Grupo CATALAD, Instituto de Química, Universidad de Antioquia, Medellín 050010, Colombia; [carlos.ostos@udea.edu.co](mailto:carlos.ostos@udea.edu.co) (C.O.)

^3^McGowan Institute for Regenerative Medicine, University of Pittsburgh, 450 Technology Drive, Suite 300, Pittsburgh, PA 15219, USA. [kims23@upmc.edu](mailto:kims23@upmc.edu) (K.S); [and78@pitt.edu](mailto:and78@pitt.edu) (A.D); [wagnerwr@upmc.edu](mailto:wagnerwr@upmc.edu) (W.R.W)

^4^Department of Congenital Heart Disease and Cardiovascular Surgery, Fundación CardioInfantil Instituto de Cardiología, Cl. 163a #13B-60, Bogotá DC 1113111, Colombia. [jbriceno@uniandes.edu.co](mailto:jbriceno@uniandes.edu.co) (J.C.B) *****

**Correspondence:**Juan C. Briceño
[jbriceno@uniandes.edu.co](mailto:jbriceno@uniandes.edu.co).
(+571) 3394949 Ext. 1758

# Supplementary Data


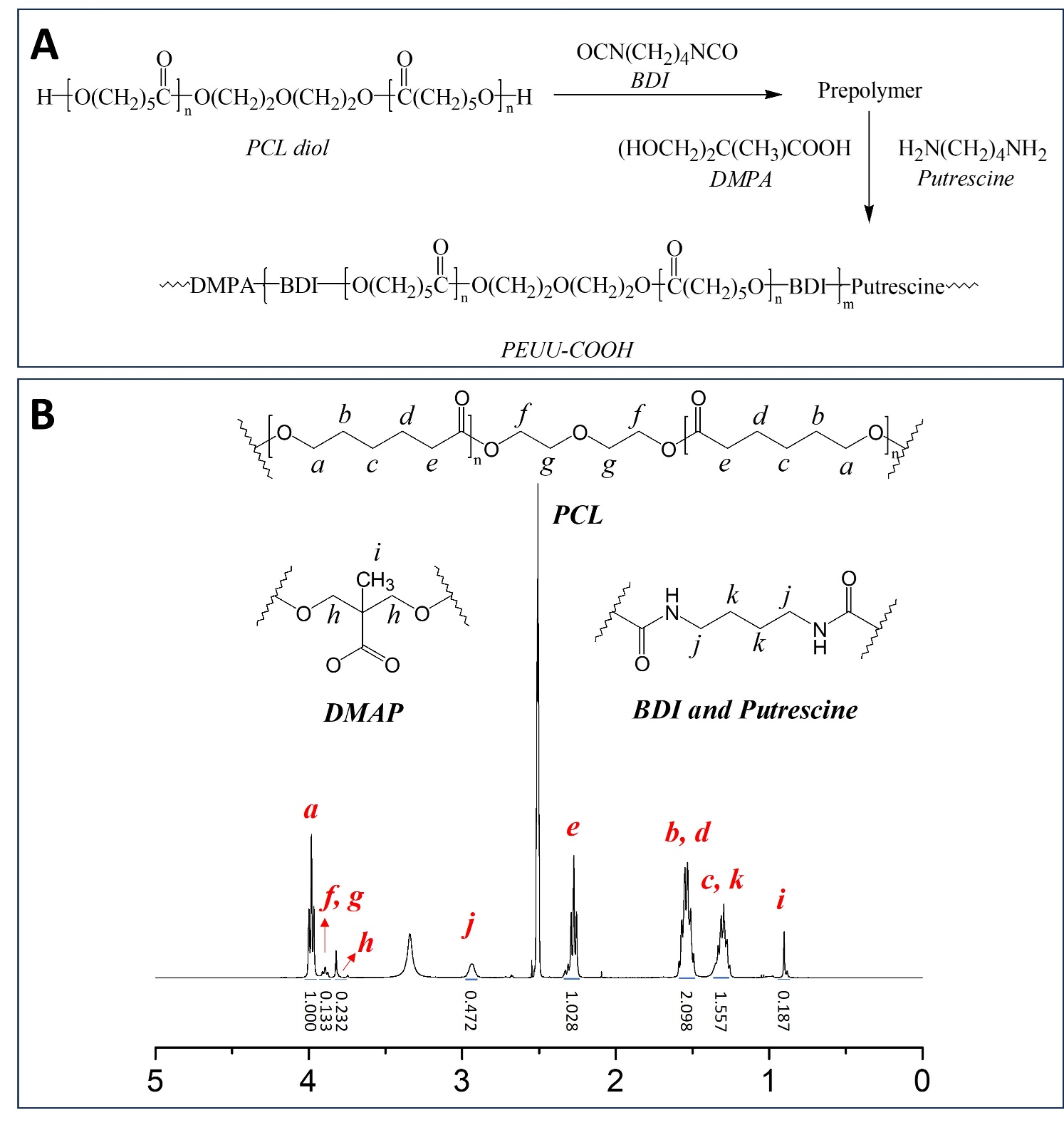


**Supplementary Figure 1.** (A) Schematic representation of the PEUU structure and PEUU-COOH synthesis, (B) ^1^H-NMR spectrum of PEUU-COOH


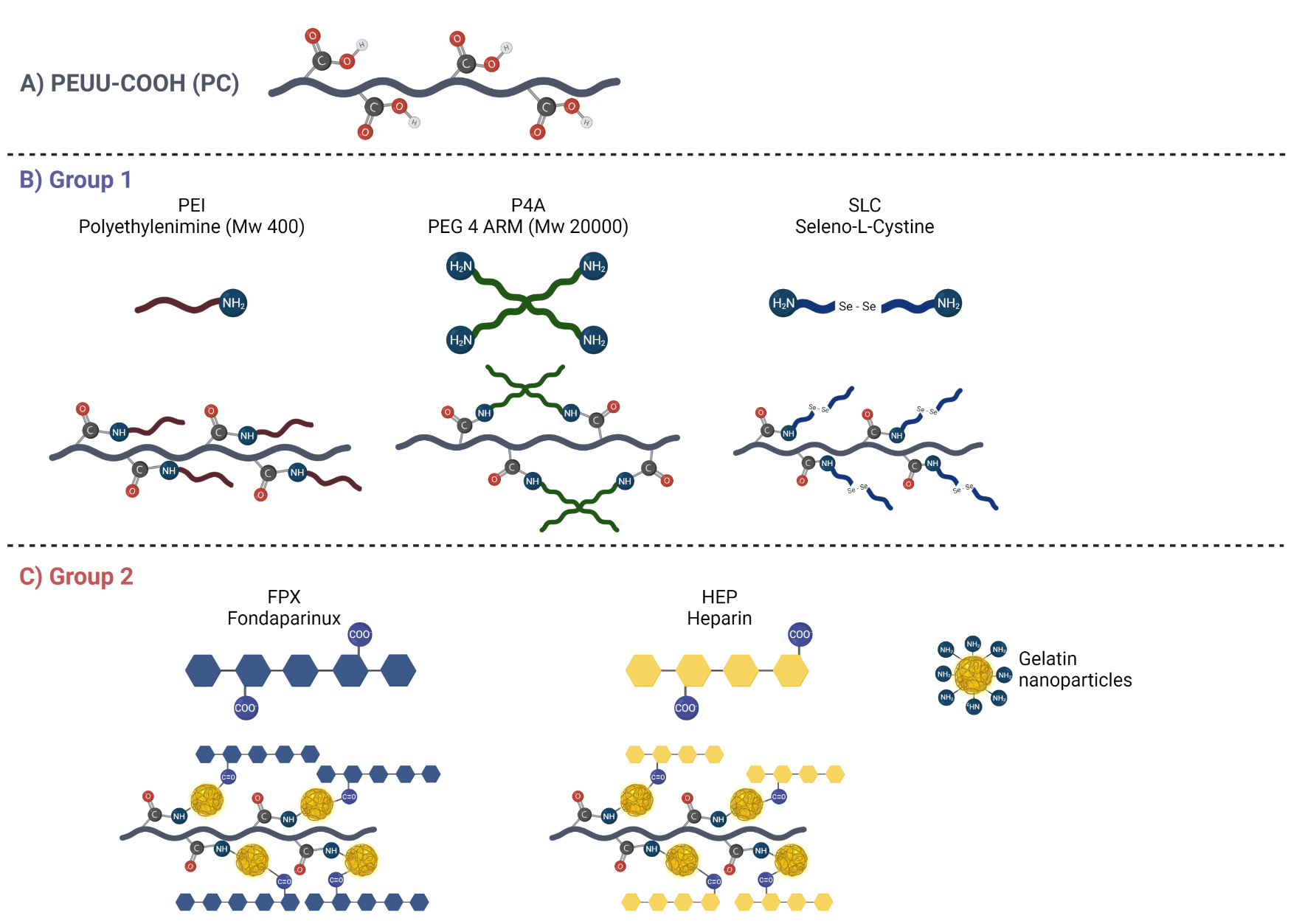


**Supplementary Figure 2.** Schematic representation of the PEUU-COOH Functionalization. A) PEUU-COOH. B) Group 1 – Direct Functionalization. C) Group 2 –Indirect Functionalization

**Table 1.** Summary of the binding energies (B.E.), full width at half maximum (FWHM), and area under the curve of XPS sub-peaks derived from PEUU Carboxylation (PC) and PC functionalization samples (Group 1 and 2). The sub-peaks are labeled in ascending order of binding energy. The resolution of the equipment used is approximately 0.5 eV. The intensity counts for the area under the curve are arbitrary and have been normalized to C1s (284.6 eV).

| Sample | Main peak | Sub-peaks | | | |
| --- | --- | --- | --- | --- | --- |
|  |  | Label | B.E. (eV) | FHWM | Area (a.u.) |
| PC Carboxylation | | | | | |
| PEUU  (reference) | C1s | 1  2  3  4  5  6 | 283.49  284.37  285.02  286.28  287.75  288.73 | 1.12  1.13  1.18  1.27  1.12  1.05 | 18.4  45.0  40.5  15.0  4.4  11.1 |
|  | O1s | 1  2  3 | 530.76  531.77  533.06 | 1.50  1.54  1.36 | 25.6  77.9  29.5 |
|  | N1s | 1  2  3 | 398.37  399.12  399.84 | 0.97  1.00  1.08 | 1.3  3.7  1.4 |
|  | | | | | |
| PC | C1s | 1  2  3  4  5  6 | 283.49  284.42  285.03  286.31  287.96  288.80 | 1.29  1.18  1.35  1.21  1.17  0.97 | 29.0  100.0  66.0  20.5  6.9  14.5 |
|  | O1s | 1  2  3 | 530.78  531.68  532.89 | 1.40  1.44  1.32 | 36.7  95.8  49.1 |
|  | N1s | 1  2  3 | 398.37  399.22  400.16 | 1.14  1.21  1.10 | 2.0  6.4  1.9 |
|  | | | | | |
| PC Functionalization | | | | | |
| Group 1 | | | | | |
| PEI | C1s | 1  2  3  4  5  6 | 283.32  284.24  285.04  285.95  287.10  288.56 | 1.39  1.11  1.21  1.32  1.25  1.29 | 28.6  75.8  70.4  53.1  9.6  13.3 |
|  | O1s | 1  2  3 | 530.73  531.65  532.68 | 1.39  1.49  1.62 | 35.5  113.4  75.7 |
|  | N1s | 1  2  3  4 | 398.40  399.17  400.01  401.37 | 0.83  1.05  1.44  1.33 | 3.0  5.8  3.7  0.8 |
|  | | | | | |
| P4A | C1s | 1  2  3  4  5  6 | 283.45  284.27  285.01  286.07  287.63  288.68 | 0.97  0.95  1.06  1.14  1.23  0.89 | 20.4  64.2  60.8  31.7  12.2  10.6 |
|  | O1s | 1  2  3 | 530.68  531.75  532.87 | 1.43  1.38  1.30 | 45.8  60.4  36.9 |
|  | N1s | 1  2  3 | 398.29  398.94  399.70 | 1.46  1.18  1.20 | 5.5  12.6  5.1 |
|  | | | | | |
| SLC | C1s | 1  2  3  4  5  6 | 283.62  284.18  285.14  286.04  287.54  288.54 | 0.87  0.95  1.06  1.21  1.38  1.03 | 24.5  75.2  58.3  31.5  24.2  16.3 |
|  | O1s | 1  2  3 | 530.66  531.59  532.79 | 1.22  1.30  1.29 | 68.1  77.7  45.6 |
|  | N1s | 1  2  3  4 | 398.21  398.98  399.70  401.12 | 1.11  1.07  1.10  1.39 | 13.7  30.2  9.7  6.0 |
|  | | | | | |
| PC Functionalization | | | | | |
| Group 2 | | | | | |
| Gelatin (reference) | C1s | 1  2  3  4  5  6 | 283.48  284.35  285.16  286.09  287.18  288.09 | 1.20  1.14  1.16  1.21  1.24  1.23 | 11.2  25.4  23.6  14.5  9.1  6.3 |
|  | O1s | 1  2  3 | 530.94  531.88  532.88 | 1.43  1.42  1.54 | 21.4  30.8  12.5 |
|  | N1s | 1  2  3 | 398.49  399.24  399.95 | 1.16  1.11  1.13 | 5.7  12.0  7.5 |
|  | | | | | |
| FPX | C1s | 1  2  3  4  5  6 | 283.75  284.52  285.24  286.18  287.78  288.78 | 1.02  0.95  1.00  1.21  1.33  1.04 | 30.9  79.6  54.6  30.9  16.2  15.5 |
|  | O1s | 1  2  3 | 531.11  531.90  533.04 | 1.25  1.32  1.35 | 54.2  73.2  47.8 |
|  | N1s | 1  2  3 | 398.31  399.13  400.78 | 0.89  1.05  1.13 | 4.1  16.2  8.7 |
|  | | | | | |
| HEP | C1s | 1  2  3  4  5  6 | 283.43  284.31  285.13  286.17  287.25  288.68 | 1.24  1.18  1.23  1.36  1.51  1.24 | 21.7  5.7  51.0  24.4  10.0  12.3 |
|  | O1s | 1  2  3 | 531.13  532.02  533.03 | 1.28  1.32  1.37 | 50.7  50.1  30.1 |
|  | N1s | 1  2  3 | 398.50  399.24  400.06 | 1.04  0.95  1.00 | 5.2  7.7  4.8 |
